# A Role for the Long Noncoding RNA *SENCR* in Commitment and Function of Endothelial Cells

**DOI:** 10.1038/mt.2016.41

**Published:** 2016-04-05

**Authors:** Mounia Boulberdaa, Elizabeth Scott, Margaret Ballantyne, Raquel Garcia, Betty Descamps, Gianni D Angelini, Mairi Brittan, Amanda Hunter, Martin McBride, John McClure, Joseph M Miano, Costanza Emanueli, Nicholas L Mills, Joanne C Mountford, Andrew H Baker

**Affiliations:** 1 Institute of Cardiovascular and Medical Sciences, BHF Glasgow Cardiovascular Research Centre, University of Glasgow, Glasgow, UK; 2 BHF/University Centre for Cardiovascular Science, University of Edinburgh, Edinburgh, UK; 3 Bristol Heart Institute, School of Clinical Sciences, University of Bristol, Bristol, UK; 4 National Heart and Lung Institute, Imperial College London, London, UK; 5 Aab Cardiovascular Research Institute, University of Rochester School of Medicine and Dentistry, New York, New York, USA; 6 Research, Development & Innovation, Scottish National Blood Transfusion Service, Ellen's Glen Road, Edinburgh, UK

## Abstract

Despite the increasing importance of long noncoding RNA in physiology and disease, their role in endothelial biology remains poorly understood. Growing evidence has highlighted them to be essential regulators of human embryonic stem cell differentiation. *SENCR*, a vascular-enriched long noncoding RNA, overlaps the Friend Leukemia Integration virus 1 (*FLI1*) gene, a regulator of endothelial development. Therefore, we wanted to test the hypothesis that *SENCR* may contribute to mesodermal and endothelial commitment as well as in endothelial function. We thus developed new differentiation protocols allowing generation of endothelial cells from human embryonic stem cells using both directed and hemogenic routes. The expression of *SENCR* was markedly regulated during endothelial commitment using both protocols. *SENCR* did not control the pluripotency of pluripotent cells; however its overexpression significantly potentiated early mesodermal and endothelial commitment. In human umbilical endothelial cell (HUVEC), *SENCR* induced proliferation, migration, and angiogenesis. *SENCR* expression was altered in vascular tissue and cells derived from patients with critical limb ischemia and premature coronary artery disease compared to controls. Here, we showed that *SENCR* contributes to the regulation of endothelial differentiation from pluripotent cells and controls the angiogenic capacity of HUVEC. These data give novel insight into the regulatory processes involved in endothelial development and function.

## Introduction

Proangiogenic therapy to promote regeneration of damaged tissue is a challenge for medicine. Human embryonic stem cells (hESC) offer a broad perspective to regenerative medicine due to their ability of self-renewal and differentiation into the three germ layers including mesoderm.^1^ Mesodermal commitment is dependent on epithelial to mesenchymal transition (EMT) described by the loss of epithelial marker CD326 and the acquisition of the neural cell adhesion molecule CD56.^2^ The mesodermal specification is an important step in vascular development in controlling the *de novo* emergence of primordial endothelial cells (EC).^3^ The potential for hESC to form endothelium has been studied using 2D monolayer-directed differentiation or 3D embryoid body (EB) differentiation culture.^4^ However, a specific developmental stimulus sufficient to support the specification of large numbers of functional EC from hESC (hESC-EC) is poorly understood. Accordingly, the comprehension of the molecular mechanisms governing hESC fate is a crucial step for their clinical use in cell-based therapies.

Noncoding RNAs (ncRNAs) have emerged to be essential regulators of cell function and identity. NcRNAs are classified by size and include long ncRNAs (lncRNA, >200 nucleotides) and small ncRNAs (<200 nucleotides) such as microRNA (miRNA). MiRNA play an important role in maintaining pluripotent state and stimulating differentiation of hESC including EC commitment.^5^ LncRNA participate in a variety of biological processes, such as chromosome imprinting, epigenetic regulation, and reprogramming of induced pluripotent stem cells.^6^ Recently, functional lncRNA have emerged as novel regulators of hESC pluripotency and differentiation.^7^ However, the role of lncRNA in mesodermal and EC commitment and function is poorly understood. Recently, *FENDRR* and *BRAVHEART* have been described as mesodermal-specific lncRNA and shown to be important for heart development.^8,9^ *TIE1-AS* was the first lncRNA described as an essential modulator of EC development and altered in patients with vascular malformation.^10^ *ALIEN* and *PUNISHER* have been identified to be orchestrators of cardiovascular commitment and EC function, respectively.^11^ Overall, these reports highlight the importance of lncRNA in the control of cell lineage specification during embryonic development.

*SENCR* is a human vascular-enriched lncRNA located on the chromosome 11 and existing as 2 isoforms including a full length (*SENCR V1*) and an alternative spliced variant (*SENCR V2*)^12^ (**Supplementary Figure S1**). *SENCR* has been described to stabilize the differentiated state of human vascular smooth muscle cells (VSMC) and is transcribed antisense from the first intron of the Friend Leukemia Integration virus 1 (*FLI1*) gene.^12^ *FLI1* is a member of E26 Transformation-specific (ETS) family expressed in the first step of EC development and is one of the earliest expressed transcription factors (TF) involved in EC specification.^13^

Since *SENCR* was found to be most abundant in EC and to correlate with expression of *FLI1*,^12^ we hypothesized that it may play a role during EC differentiation and in EC function. To investigate this, we developed two novel protocols allowing efficient EC differentiation from hESC using direct and haemogenic routes. We then profiled the dynamic regulation of *SENCR* during endothelial differentiation. We further characterized its role in controlling the mesodermal and endothelial commitment of hESC. Finally, we revealed an alteration of *SENCR* in vascular tissue and EC derived from patients with critical limb ischemia and premature coronary artery disease.

## Results

### Differentiation protocols using direct routing or hemogenic endothelium formation allow generation of EC from hESC

**Figure 1:**
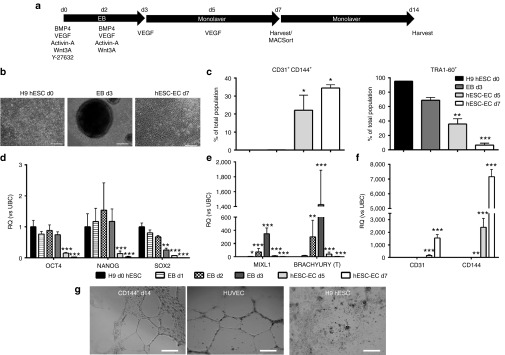
**Direct differentiation of human embryonic stem cells (hESC) to endothelial cell (EC)**. (**a**) Schematic representation of direct hESC-EC differentiation. (**b**) Morphological analysis of H9 hESC, d3 EB, and d7 hESC-EC (scale bars 250 µm). (**c**) Fluorescence-activated cells sorting quantification for CD144/CD31 (left) and TRA1-60 (right) in H9 hESC, d3 EB, and d5-7 hESC-EC (*n* = 3, error bars = standard error of the mean). (**d–f**) Quantitative reverse transcription-PCR analysis of pluripotency (**d**), mesodermal (**e**), and endothelial (**f**) genes (*n* = 4, error bars = RQ_max_). (**c–f**) Repeated measures analysis of variance, Tukey's *post-hoc* comparisons, *P < 0.05, **P < 0.01, ***P < 0.001 compared to d0. (**g**) Tubule formation analysis of d14 CD144^+^CD31^+^ hESC-EC. d0 H9 hESC was used as a negative control and human umbilical endothelial cell as a positive control. Images taken at 10× magnification (scale bars represent 100 µm). *n* = 3; images are representative for sample group.

**Figure 2:**
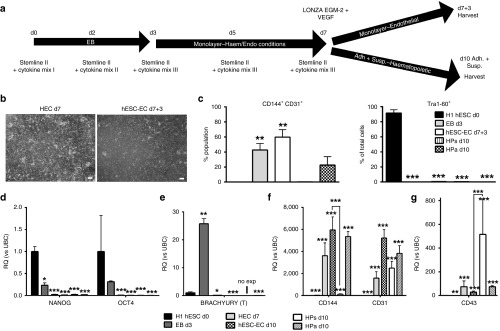
**Indirect endothelial cell (EC) differentiation formation of embryonic stem cells (HEC)**. (**a**) Schematic representation of indirect hESC-EC differentiation. (**b**) Morphological analysis of d7 HEC and d7+3 hESC-EC (scale bars 250 µm). (**c**) Quantification of fluorescence-activated cells sorting for both CD144^+^CD31^+^ (left) and TRA1-60^+^ (right) in H1 hESC, d3 EB, d7 HEC, d7+3 hESC-EC, d10 HPs, and d10 HPa. (*n* = 3, error bars = SEM). (**d–g**) Quantitative analysis of pluripotent (**d**), mesodermal (**e**), endothelial (**f**), and hematopoietic (**g**) genes (*n* = 3, error bars = RQ_max_). (**c–i**) Repeated measures analysis of variance, Tukey's *post-hoc* comparisons, *P < 0.05, **P < 0.01, ***P < 0.001 compared to d0 H1 hESC pluripotent control.

To investigate the role of lncRNA during EC development, we established two novel protocols for the derivation of hESC-EC; direct and indirect, the latter routed through hemogenic endothelium. For direct hESC-EC differentiation, H9 d0 hESC were forced into EB, plated out on d3 and cultured for 4 days in EGM-2 (**Figure 1a**,**b**). At d7, EC markers CD144 and CD31 were increased, while pluripotency-marker TRA1-60 was decreased (**Figure 1c**, **Supplementary Figure S2a** top). Pluripotency-associated genes *OCT4, NANOG*, and *SOX2* were decreased from d3 (**Figure 1d**), mesodermal genes *BRACHYURY* and *MIXL1* peaked at d3 (**Figure 1e**), and EC genes *CD31* and *CD144* were upregulated from d3 (**Figure 1f**). Similar results were obtained using H1 hESC line (**Supplementary Figure S2a** bottom, **Supplementary Figure S2b**). D7 hESC-EC CD144^+^ and CD144^-^ were purified and expanded for 7 days, then expression of EC markers CD144 and CD31 was analyzed (**Supplementary Figure S3**). After 7 days of expansion, CD144^+^ hESC-EC, coexpressing CD31, were able to form tube-like structures on matrigel (**Figure 1g**) supporting their functionality. Hemogenic endothelial cells (HEC) are progenitors, hypothesised to give rise to EC and hematopoietic progenitor cells (HP) during development,^14^ previously identified as CD144^+^CD73^−^CD235a^−^CD43^−^CD117^intermediate^.^15^ EC generation through HEC was developed using the first 7 days of an HP differentiation protocol (**Supplementary Methods**, **Figure 2a**). By d7, we observed the formation of suspension HP (HPs), which bud off adherent HP (HPa). Within HPa cells, we identified a CD31^+^CD144^+^CD235A^−^CD117^−^CD43^mixed^CD73^mixed^ population with cells expressing either CD43 or CD73 (**Supplementary Figure S4**), similar to previously described for HEC.^15^ EC capacity of d7-9 HPa was examined by culturing d7, 8 or d9 HPa cells in EGM-2 medium containing FBS for 3, 2 or 1 day until d10 (hESC-EC d7+3, d8+2, d9+1 respectively). Generation of hESC-EC was efficient using 3 days in EC culture media (hESC-EC d7+3) (**Supplementary Figure S5**). HESC-EC d7+3 formed confluent monolayers, with a reduction in budding and suspension cells (**Figure 2b**). HEC were cultured in HP and EC conditions to compare their EC and hematopoietic capacities (**Figure 2a**). The percentage of CD144^+^CD31^+^ cells was significantly higher in the hESC-EC d7+3 compared to d7 HEC and d10 HPs/a, and a decrease in TRA1-60^+^ cells was observed throughout differentiation (**Figure 2c**). Moreover, pluripotent genes *NANOG* and *OCT4* were downregulated from d3, and *BRACHYURY* expression peaked at d3 (**Figure 2d**,e). The EC-associated gene, *CD144* was upregulated in d7 HEC, d10 HPa, in hESC-EC d7+3, and was observed only at very low levels in d10 HPs (**Figure 2f**). *CD31* was upregulated in all populations after d3, and its expression has been associated with both EC and HP cells (**Figure 2f**). *CD43*, a HP-associated gene, was more highly expressed in d10 HPs compared to d7 HEC and hESC-EC d7+3 (**Figure 2g**).

### CD326^low^CD56^high^ mesoderm progenitor populations during hESC-EC differentiation

Using direct and HEC routes, a population of mesodermal progenitor (MP) appeared transiently at d3 and were identified as CD326^low^CD56^high^ as previously described^2^ (**Supplementary Figure S6**). CD326^low^CD56^high^ d3 population was isolated using fluorescence-activated cells sorting after direct EC differentiation (**Supplementary Figure S7a**) and gene profile was analysed (**Supplementary Figure S7b**). This data supports emergence of early MP identified as CD326^low^CD56^high^. These data suggest that these two protocols were able to efficiently generate CD144^+^CD31^+^ hESC-EC with a consistent EC phenotype allowing us to interrogate the role of *SENCR* in EC development.

### *SENCR* is dynamically regulated in endothelial and erythroid derivatives of hESC

**Figure 3:**
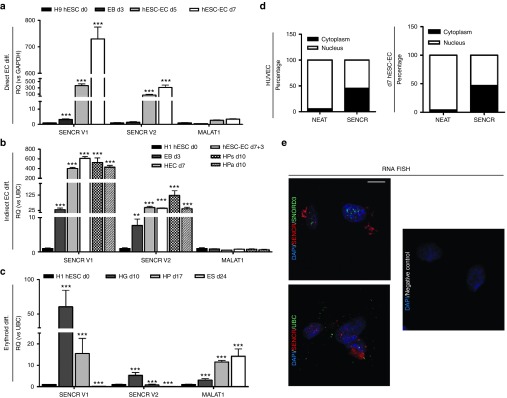
**Regulation of *SENCR* in human embryonic stem cells (hESC) derivatives**. (**a–c**) Histograms represent expression of *SENCR* V1, *SENCR V2*, and *MALAT1* during: (**a**) Direct EC differentiation versus H9 hESC compared to d3 EB, d5-7 hESC-EC; (**b**) Indirect differentiation of hESC into endothelial cells (EC) versus H1 hESC compared to d3 EB, d7 HEC, d7+3 hESC-EC, d10 HPa/s; (**c**) Erythroid differentiation versus H1 hESC compared to d10 haemangioblast (HG), d17 HP, d24 erythroid cells (ES). (**a–c**) Repeated measures analysis of variance, Tukey's *post-hoc* comparisons, *n* = 3, *P < 0.05, **P < 0.01, ***P < 0.005 compared to d0 (all error bars = RQ_max_). (**d**) Subcellular localization of *SENCR* and *NEAT1* in human umbilical endothelial cell (HUVEC) (left) and d7 hESC-EC (right) using RNA fractionation. The equal volumes of nuclear and cytoplasmic RNA, corresponding to equivalent numbers of cells were used for cDNA synthesis. The abundance of transcript in nuclear and cytoplasmic fraction was analysed by quantitative reverse transcription-PCR and normalized using UBC. (**e**) RNA FISH analysis targeting *SENCR* (red) versus *UBC* (green) and nuclear *SNORD3* (green) in HUVEC (scale bars 25 µm).

Because *SENCR* is enriched in EC and correlates with expression of *FLI1*,^12^ a regulator of EC development,^13^ we first quantified *SENCR* expression during EC differentiation using our protocol. We also profiled *MALAT1* expression which is known to regulate EC function.^16^ During direct hESC-EC differentiation (**Figure 3a**), *SENCR V1* but not *V2* was upregulated at d3 and sustained in hESC-EC at d5 and 7. *SENCR V2* and *MALAT1* were upregulated at d5 and increased at d7. *SENCR V1* was consistently induced to a higher level than *SENCR V2* and *MALAT1*. During generation of EC through HEC (**Figure 3b**), *SENCR V1* and *V2* were induced from d3 and upregulated in d7 HEC, d7+3 hESC-EC, and d10 HPa/s. *SENCR V1* was still the more highly induced variant. *MALAT1* was not modulated. During erythroid differentiation (**Figure 3c**), *SENCR V1* was the higher expressed lncRNA in d10 haemangioblast. In d17 HP, *SENCR V1* and *V2* were silenced while *MALAT1* remained constantly upregulated. In d24 erythroid cells, *SENCR V1* and *V2* were switched off when *MALAT1* was stably expressed. We concluded that *SENCR V1* was consistently and highly upregulated in early endothelial progenitor and remained expressed in hESC-EC. As *SENCR V1* (hereafter called *SENCR*) was the more abundant variant in hESC-EC, we focused on its role in differentiation. Since *FLI1* is the antisense gene of *SENCR*, we analysed any correlation between *FLI1* and *SENCR* during differentiation. Consistent with previous findings in mature vascular cells,^12^ expression of *SENCR* and *FLI1* was positively correlated during EC and HP differentiation (**Supplementary Figure S8**). Time course expression demonstrated that *FLI1* and *SENCR* were activated at d1 and were then constantly upregulated during EC differentiation and in human umbilical endothelial cell (HUVEC)(**Supplementary Figure S9a**). Expression of *ETV2*, an ETS factor upstream of *FLI*1,^17^ peaked at d5 then was silenced in d7 hESC-EC and in HUVEC (**Supplementary Figure S9a**). However, *ETV2* was more highly expressed compared to *SENCR* and *FLI1* in H9 hESC and in d1-2 EB (**Supplementary Figure S9b**). As cellular localisation is crucial to understand the potential function of lncRNA, we investigated expression of *SENCR* in intracellular compartment. We found an equal distribution of *SENCR* between both nucleus and cytoplasm whereas *NEAT* exhibited a high selectivity for nuclear localisation in HUVEC and hESC-EC (**Figure 3d**). RNA FISH experiments confirmed the equal distribution of *SENCR* in nucleus and cytoplasm in HUVEC compared to *UBC* and nuclear *SNORD3* (**Figure 3e**)

### *SENCR* overexpression does not impact on pluripotency

**Figure 4:**
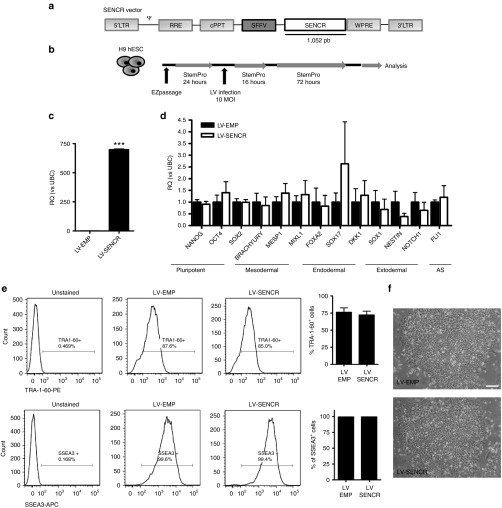
***SENCR* overexpression does not impact on human embryonic stem cells (hESC) pluripotency**. (**a**) Schematic representation of constructed recombinant lentivirus (LV) vectors harbouring *SENCR* sequence under SFFV promoter. LTR, long-terminal-repeat; Ψ = Psi, packaging signal; RRE, Rev response element; cPPT, central polypurine tract; SFFV, promoter of spleen focus-forming virus; wPRE, Woodchuck hepatitis virus posttranscriptional regulatory element. (**b**) Experimental design used to transduce pluripotent H9 hESC. (**c–f**) All of measures were performed 3 days postinfection and LV-*SENCR*-infected group is compared to LV-control infected group. (**c,d**) Gene analysis for *SENCR* expression (**c**) and pluripotent/germ-layer genes (**d**) by quantitative reverse transcription-PCR (all error bars = RQ_max_). (**e**) Representative fluorescence-activated cells sorting and quantification of immunophenotype profile analysis for TRA-1-60 and SSEA-3 (all error bars = SEM). (**f**) Image representing morphology of H9 hESC colony (scale bar 125 μm). (**a–f**) *n* = 3, Student's *t*-test. ***P < 0.005.

To assess whether *SENCR* may impact on hESC pluripotency, we used self-inactivating vesicular stomatitis virus-pseudotyped lentivirus (LV) vectors harbouring the *SENCR* sequence under the *SFFV* promoter (**Figure 4a**). *SENCR* overexpression by LV particles (**Figure 4b**,c) did not alter: the pluripotent and germ-layer gene expression profile of hESC (**Figure 4d**); the expression of pluripotency-associated surface markers TRA-1-60 and SSEA3 (**Figure 4e**); and the colony shape of hESC (**Figure 4f**). These data revealed that *SENCR* overexpression was not sufficient to drive hESC to exit their pluripotent state.

### *SENCR* overexpression potentiates the mesodermal and endothelial commitment of hESC

**Figure 5:**
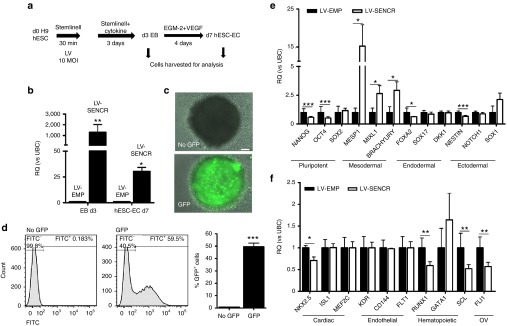
***SENCR* overexpression during EB formation induces mesodermal genes expression**. (**a**) Experimental design allowing *SENCR* overexpression during EB formation. (**b**) *SENCR* expression analysis by quantitative reverse transcription-PCR 3 and 7 days post-transduction (all error bars = RQ_max_). (**c–f**) All of measures were performed 3 days postinfection. (**c,d**) Fluorescence microscopy (**c**, scale bar 125 µm) and quantification by fluorescence-activated cells sorting of GFP^+^ cells (**d**) in EB infected with or without LV-GFP (all error bars = SEM). (**e,f**) quantitative reverse transcription-PCR analysis showing the expression of pluripotent, mesodermal, endodermal, ectodermal (**e**), cardiac, endothelial, hematopoietic and *FLI1* overlapping gene (OV) (**f**) in LV-*SENCR* group compared to LV-control (all error bars = RQ_max_). (**a–f**) *n* = 5, Student's *t*-test, *P < 0.05, **P < 0.01, ***P < 0.005.

**Figure 6:**
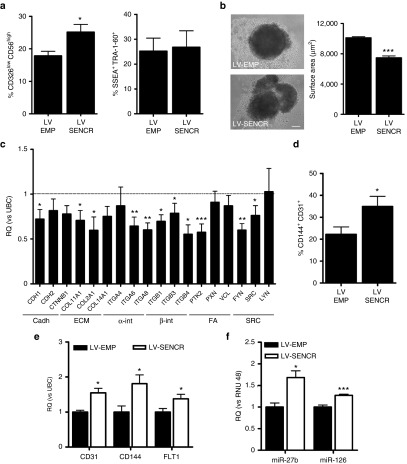
***SENCR* overexpression potentiates the mesodermal and endothelial commitment of human embryonic stem cells**. (**a–c**) All of measures were performed 3 days postinfection and LV-*SENCR* group is compared to LV-control group. (**a**) Histograms represent fluorescence-activated cells sorting analysis for MP CD326^low^CD56^high^ (left) and pluripotent marker TRA1-60^+^SSEA3^+^ (right). (**b**) Measure of area of EB by ImageJ expressed in µm^2^ (scale bar 125µm) (all error bars = SEM). (**c**) Custom Taqman low-density array analysis of stem cells adhesion genes expression including cadherins (cadh), extracellular matrix (ECM), α-integrins (α-int), β-integrins (β-int), focal adhesion (FA), and Src-Kinase (SRC) (*n* = 5, all error bars = RQ_max_). (**d–f**) All measures were performed 7 days postinfection and LV-*SENCR* group is compared to LV-control group. (**d**) Histogram representation of immunophenotype profiles of cells expressing endothelial cell markers CD144 and CD31 (*n* = 3, all error bars = SEM). (**e,f**) Endothelial genes (**e**) and miRNA (**f**) expressions analysed by quantitative reverse transcription-PCR (*n* = 3, all error bars = RQ_max_). (**a–f**) Student's *t*-test, *P < 0.05, **P < 0.01, ***P < 0.005.

To understand whether the modulation of *SENCR* may regulate hESC-EC differentiation, we developed a transduction protocol allowing *SENCR* overexpression during EB development (**Figure 5a**). Toxicity of LV infection was evaluated by flow cytometry (**Supplementary Figure S10**). *SENCR* was overexpressed 3 days postinfection and was also stably overexpressed 7 days after transduction (**Figure 5b**). LV infection using vectors carrying the green fluorescence protein (*GFP*) under control of the *SFFV* promoter demonstrated an efficient GFP expression in EB 3 days postinfection (**Figure 5c**,d). *SENCR* overexpression reduced the expression of pluripotent (*NANOG*, *OCT4*), endodermal (*FOXA2)*, and ectodermal genes (*NESTIN)* but enhanced the expression of mesodermal genes (*MESP1*, *MIXL1*, *BRACHYURY*) (**Figure 5e**). Specific cardiac (*NKX2.5*), hematopoietic (*RUNX1*, *SCL*), and *FLI* genes were reduced while EC genes were unchanged (**Figure 5f**). Since exogenous *SENCR* enhanced mesodermal gene expression, we then investigated whether *SENCR* drives MP commitment. An increase in CD326^low^CD56^high^ MP was observed 3 days after *SENCR* overexpression while pluripotency-associated markers SSEA-3 and TRA1-60 were unchanged (**Figure 6a**) (**Supplementary Figure S11a,b**). EB overexpressing *SENCR* displayed a less compact and more irregular shape as well as a reduction in surface area (**Figure 6b**). EMT, characterized by a loss of epithelial gene such as *CDH1*, is a process governing the emergence of MP^18^ and we therefore wanted to understand the mechanism involved in this phenotype. A custom TaqMan Low Density Array identified stem cell adhesion molecules that were downregulated in EB overexpressing *SENCR* including *CDH1* (**Figure 6c**). We found also that *SENCR* overexpression after 7 days, stimulated generation of hESC-EC expressing CD144^+^CD31^+^ (**Figure 6d**) (**Supplementary Figure S11c**) as well as increased expression of EC-genes (*CD31*, *CD144*, *FLT1)* and EC-miRNA (*miR-27b*, *miR-126*) (**Figure 6e**,f). Collectively, these data revealed the potential role of *SENCR* in controlling the EC commitment from hESC through MP.

### *SENCR* regulates proliferation, migration, and tube-like formation of HUVEC

**Figure 7:**
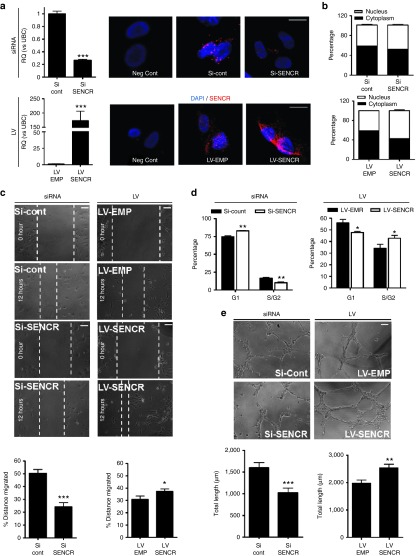
***SENCR* controls proliferation, migration and tube like formation in human umbilical endothelial cell (HUVEC)**. (**a**) *SENCR* expression analysis by quantitative reverse transcription-PCR and RNA FISH (scale bar 25 µm) in HUVEC subjected to siRNA-Pool transfection targeted or not against *SENCR* (top) and to LV infection (bottom) (all error bars = RQ_max_). (**b**) Subcellular localization using RNA fractionation of *SENCR* in HUVEC subjected to siRNA transfection (top) and LV infection (bottom). (**c**) Following siRNA transfection (left) or LV infection (right) HUVEC were starved for 12 hours and mechanically disrupted with a sterile 200 μl tip, then photographed immediately at 0 and 12 hours latter. Migrated distance was measured as percentage by ImageJ and representative images are shown (scale bar 125 µm). (**d**) Percentage of cells in G0/G1 and S/G2 measured by flow cytometry following siRNA transfection (left) and LV infection (right). (**e**) Following siRNA transfection (left) or LV infection (right) HUVEC were starved for 12 hours and were allowed to sprout in a three-dimensional matrix for 12 hours. Cumulative sprout length was quantified using angiogenesis analyser for ImageJ and representative images are shown (scale bar 125 µm) (all error bars = SEM). (**a–d**) *n* = 3, Student's *t*-test, *P < 0.05, **P < 0.01, ***P < 0.005.

Because *SENCR* expression remained elevated in hESC-EC, we investigated the role of *SENCR* in HUVEC. We first elucidated the response of *SENCR* expression in HUVEC subjected to treatment with vascular endothelial growth factor (VEGF), a cytokine involved in biological activity of EC.^19^ VEGF stimulated HUVEC proliferation (**Supplementary Figure S12a**) and induced *SENCR* expression transiently and in a time dependant manner with a peak 12 hours after stimulation (**Supplementary Figure S12b**) demonstrating its role in physiology of EC. To understand the role of *SENCR* in EC, loss- and gain-of-function studies were performed in HUVEC transfected with siRNA-Pool or infected with LV, respectively. *SENCR* knockdown and overexpression were confirmed by RNA FISH and quantitative reverse transcription-PCR (**Figure 7a**). Moreover siRNA and LV did not impact on cellular localization of *SENCR* (**Figure 7b**). We examined the ability of *SENCR* to affect cell motility using the scratch “wound” healing assays in HUVEC. Silencing and overexpression of *SENCR* reduced and stimulated, respectively, the covering of wounded area after 12 hours suggesting that *SENCR* promotes EC migration (**Figure 7c**). Cell cycle was next analysed to understand whether *SENCR* modulation may cause cell cycle arrest. *SENCR* silencing led to an increase of cells in the G1 phase supported by a decrease of cells in the S/G2 phase whereas its overexpression had the opposite effect (**Figure 7d**). Since migration and proliferation are important in angiogenesis, we focused on the impact of *SENCR* modulation in this process. Knockdown and overexpression of *SENCR* impaired and stimulated, respectively, capillary-like structures on matrigel (**Figure 7e**).

### *SENCR* controls the expression of angiogenic-related genes in HUVEC

**Figure 8:**
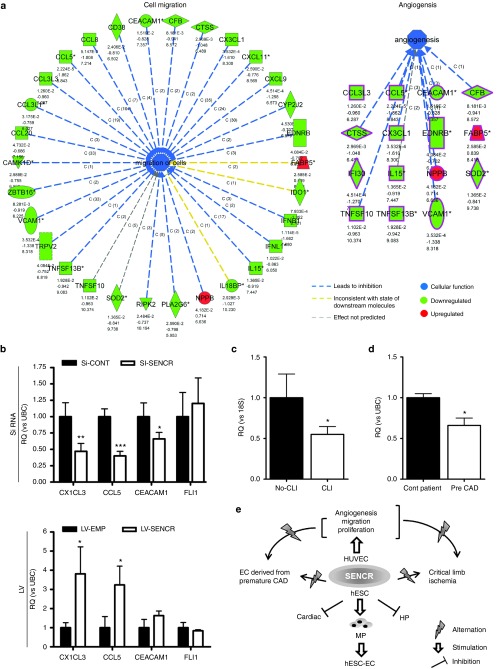
***SENCR* controls the expression of angiogenic-related gene and is altered in human sample derived from ischemic condition**. (**a**) These figures illustrate genes with expressional changes after *SENCR* silencing belong to the cell migration (left) and angiogenesis (right). Gene expression profiles were uploaded to the Ingenuity Analysis Software and based on the differential expression of these genes, the most relevant biochemical network with functional links was assessed. (**b**) Quantitative reverse transcription-PCR was used to analyse expression of *CX1CL3*, *CCL5*, *CEACAM1* as well as *FLI1* in human umbilical endothelial cell subjected to siRNA transfection (top) and LV infection (bottom) (all error bars = RQ_max_), *n* = 3, Student's *t*-test, *P < 0.05, **P < 0.01, ***P < 0.005. (**c**) Comparison of *SENCR* expression for the critical limb ischemia group (CLI, *n* = 10, *P < 0.05) versus control group (No-CLI, *n* = 9). (**d**) Comparison of *SENCR* expression for superficial forearm vein endothelial cells derived from premature coronary artery disease (CAD) group (Pre-CAD, *n* = 8, *P < 0.05) versus control group (Cont patient, *n* = 8) (all error bars = RQ_max_). Student's *t*-test. (**e**) Schematic representation of the role of *SENCR* in hESC-EC differentiation and endothelial cell function.

To understand the targeted gene modulated by silencing of *SENCR*, we next carried out a microarray analysis using siRNA-treated cells. A schematic presentation (**Figure 8a**) shows that known migratory and angiogenic genes were downregulated after silencing of *SENCR*. We focused our study on *CCL5*, *CEACAM1* and *CX3CL1* described as proangiogenic gene.^20,21,22,23^ We confirmed that knockdown of *SENCR* resulted in a decrease of these genes and its overexpression induced an upregulation of *CCL5* and *CX3CL1* (**Figure 8b**). As shown by Bell *et al*.,^12^ modulation of *SENCR* did not alter the expression of *FLI1* in this scenario demonstrating that *SENCR* regulated EC function independently of *FLI1* (**Figure 8b**). These data revealed *SENCR* as a regulator of angiogenesis-related function in EC.

### *SENCR* dysregulation is associated with premature coronary artery disease and critical limb ischemia

Since *SENCR* regulates angiogenic-related function in HUVEC, we interrogated whether *SENCR* alteration may be associated with angiogenesis dysfunction in human samples. *SENCR* level was reduced, versus control limb muscle sample, in human critical limb ischemia (CLI) (**Figure 8c**), an occlusive peripheral arterial disease resulting in inadequate perfusion of the limb.^24^ Alterations in EC function are common to a variety of pathologies including CLI and coronary artery disease (CAD).^25^ Particularly, EC dysfunction has been associated with the premature onset of CAD.^26^ EC derived from the superficial forearm veins were previously isolated from pre-CAD patients and healthy control subjects. These cells have a high expression of mature endothelial cell antigens vWF, CD31, CD146, and KDR, and lack expression of the haematopoietic antigen, CD45.^27^ This study demonstrated impaired proliferation, adhesion, and migration of vessel wall EC derived from the superficial forearm veins in these patients compared to matched control subjects.^27^ Consistent with these observations, we found that *SENCR* expression was significantly reduced in vessel wall EC isolated from patients with premature CAD compared to control subjects (**Figure 8d**). Collectively, these data showed that *SENCR* expression is down regulated in patients with EC dysfunction and atherosclerotic vascular disease.

## Discussion

We aimed to investigate the role of *SENCR* in EC differentiation and function by using two novel differentiation protocols allowing efficient hESC-EC generation, through direct and haemogenic routes. We demonstrated that *SENCR* was regulated during hESC-EC commitment, and through gain-of-function study, we revealed *SENCR* as an early induced lncRNA capable of promoting mesodermal and EC commitment. Therefore, loss- and gain-of-function experiments identified *SENCR* as a regulator of proliferation, migration, and angiogenesis in EC. Finally, altered *SENCR* expression was found in CLI tissues and in EC derived from premature CAD (**Figure 8e**).

*SENCR* is vascular-enriched lncRNA overlapping *FLI1* gene,^12^ a member of ETS family, which has been described as an early regulator of hemato-endothelial development.^13,17,28^ ETS factors are involved in developmental processes and ETS-binding sites are not specific for EC-expressed gene loci making difficult the comprehension of mechanisms involved in ETS factor-regulating EC specification. ETS factors controlling-EC gene depend on complex processes including binding partners, post-transcriptional modification and flanking sequence content.^29^ The functional role of ETS factors in EC fate has been, for example, explained by a probable cooperative action with EC TF such as *GATA-2*.^13^ With tissue-specific expression patterns during development, lncRNA may be orchestrators of this process and some reports have already related their role in specification of germ-layer^9,11^ and adult cells.^9,10,11^ Here, we showed the dynamic regulation of a lncRNA, *SENCR,* and an overlapping ETS gene, *FLI1*, during hESC-EC differentiation in precursor and differentiated hESC-EC. These data support a previous report associating induction of *FLI1* to EC development^28^ and revealed *SENCR* as an early mesodermal lncRNA remaining expressed in hESC-EC. Addition of exogenous *SENCR* during EB formation increased the generation of MP expressing CD326^low^CD56^high^ and induced expression of mesodermal genes including *BRACHYURY*, *MIXL1*, and *MESP1*. EMT, controlling generation of MP, is mediated by TF such as *MESP1* and characterized by the loss of epithelial genes such as *CDH1*.^2^ MESP1, through EMT, drives generation of cardiovascular MP including EC and VSMC whilst inhibiting the formation of other MP lineages such as HP.^30^ *MESP1* was upregulated when *CDH1* was downregulated after *SENCR* overexpression suggesting that *SENCR* can positively manipulate cardiovascular MP specification. This correlated with the fact that EB overexpressing *SENCR* failed to form compact structures and with a report which described CDH1 as a factor of EB aggregation.^31^ Exogenous *SENCR* also induced a downregulation of extracellular matrix molecules such as *COL2A1* and of β-integrins genes such as *ITGB1,* both pathways linked to cardiac specification.^32^ Moreover, a previous study related the size of EB to efficiency of EC differentiation^33^ suggesting that EB morphology impacts on specification of vascular MP. Similarly, addition of exogenous *SENCR* resulted in a reduction in the size of EB. These demonstrated that *SENCR* may impact on the generation of vascular MP through molecular and morphological changes. *NKX2.5*, involved in cardiac development,^34^ as well as *SCL* and *RUNX1*, both involved in HP specification,^35,36^ were downregulated after *SENCR* overexpression while EC genes were unchanged reaffirming the hypothesis that *SENCR* is part of a molecular axis, driving vascular fate while inhibiting the formation of other mesodermal lineages.

Our observations revealed that exogenous *SENCR* impacts on vascular mesodermal specification, essential for EC development,^3,6^ is in concordance with the findings that *SENCR* overexpression led to an increase in CD31^+^CD144^+^ hESC-EC at d7 of differentiation. This was accompanied with an enhancement in EC-specific genes and miRNA including *CD144*, *CD31, FLT1, miR-126 and miR-27b. SENCR,* and *FLI1* shared the same expression profile throughout EC and erythroid differentiation highlighting their identical transcriptional regulation during development. To act at transcriptional level, ETS factors bind to a core “GGAA/T” binding element,^37^ and *FLI1* itself is activated by *ETV2* via an ETS binding site located -192 upstream its start site.^17^ Therefore, analysis of genomic sequences revealed an ETS binding site -149 upstream *SENCR* start site, and transcription of both *FLI1* and *SENCR* gene was activated from d1 of differentiation. Moreover, studies supported common TF activating both protein coding, and antisense lncRNA.^38^ Upon vascular development, *ETV2* expression turns off and FLI controls its own expression through an auto-regulatory loop.^17^ Consistent with these statements, expression of *ETV2* was consistently increased on the first day of differentiation and silenced in fully differentiated hESC-EC and in HUVEC. Moreover, *FLI1* and *SENCR* expression was not dependant on *ETV2* silencing at the end of differentiation. This asks the question of how *SENCR* remained expressed as well in EC. The influence of *SENCR* modulation on *FLI1* expression and *vice versa* has not yet been described^12^ eliminating cis-acting effect on each other. Our gain- and loss-of-function experiment in HUVEC confirmed this fact; however, we found that expression of exogenous *SENCR* in EB led to a reduction of *FLI1* transcription. This may be explained by the downregulation of *SCL,* induced by exogenous *SENCR*. Indeed *SCL* and *FLI1* form a regulatory circuit controlling specification of HP by maintaining expression of each other.^39^ Nevertheless, despite the reduction of *FLI1* in EB, its level seems to be sufficient to drive EC differentiation through MP in concert with exogenous *SENCR* transcript. Active transcription of *SENCR* during EC differentiation may be explained as *FLI1* by a positive auto-regulatory loop similarly to the lncRNA *HULC*, in hepatocellular carcinoma which exists as part of an intricate auto-regulatory network resulting in an increase of its own expression.^40^ Another explanation of the high levels of *SENCR* expression in hESC-EC and cultured EC may be due to the stability of the RNA after post-transcriptional modification similar to *ZFAS1* lncRNA which is extremely stable and highly expressed in mouse neuroblastoma cells.^41^

*SENCR* was initially discovered as a vascular enriched lncRNA and described to inhibit migration and stimulate contractile gene expression of VSMC.^12^ The function of *SENCR* in adult EC has not yet been studied; although its high expression in EC^12^ reflected its potential importance. Along this line, silencing and overexpression of *SENCR* impaired and enhanced, respectively, sprouting of cultured EC as well as the expression of proangiogenic genes including *CCL5*, *CEACAM-1*, and *CX1CL3*. Angiogenesis is a multistep process including a first step where EC migrate into the extracellular space, proliferate, and form capillary sprouts and tubular structures. CEACAM-1 is a transmembrane protein expressed on cell surface of capillary EC and has been described to stimulate angiogenesis through promigratory process.^23^ In correlation with these observations, the migratory capacity of EC was inhibited and stimulated after *SENCR* silencing and overexpression, respectively. Bell *et al*.^12^ described *SENCR* as a suppressor of promigratory phenotype in VSMC highlighting, its probable function in disease processes such as atherosclerosis where VSMC migration contributes to the pathogenesis of neo-intima formation. Currently, treatments for CAD use drug-eluting stents allowing the delivery of a nonspecific antiproliferative agent acting on both VSMC and EC leading to a delay of re-endothelialisation and restenosis.^42^ Ideally, treatments would inhibit the VSMC proliferation and migration, whilst promoting EC function. In this direction, *SENCR* is able to inhibit promigratory phenotype of VSMC while stimulating angiogenesis of EC similar to *miR-126* which inhibits VSMC migration while restoring EC function in animal models of vascular balloon injury.^43^ The proangiogenic and promigratory effect induced by *SENCR* on cultured EC were also correlated in limb ischemia, pathology induced by vascular injury, where *SENCR* expression was altered. Consistent with that, *CX3CL1*, downregulated after *SENCR* silencing, has been demonstrated to significantly reverse limb ischaemia in a rat model via the induction of effective revascularization.^21^ Amongst other genes, *CX3CL1* simulates tissue neovascularization through the enhancement of EC proliferative capacity. Our findings showed that *SENCR* also impacts on cell cycle progression by stimulating cells to enter S/G2 phase. *SENCR* was also impaired in vessel wall EC, derived from patient with premature CAD, displaying defects in proliferative and migratory processes. The purpose of this study was to define a novel molecular mechanism that may have an application in the development of effective hESC-EC therapies for ischemic disease. Only a few reports have described the role of lncRNA in vascular development and pathology.^29^ For example, *MALAT1* has been described to be significantly upregulated during hypoxia and controlling sprouting and migratory capacity of EC;^44^ however loss-of-function studies in mice revealed that it is not essential for development.^7^ *TIE1-AS* is the only lncRNA described to be essential for EC development and altered in human vascular injury.^10^ Here, we described for the first time an ETS-related lncRNA involved in EC development and function as well as its association with human vascular pathology.

## Materials and Methods

***Cell culture.*** The hESC lines H1 and H9 (WiCell Research Institute, Madison, WI, http://wicell.org) were cultured in a feeder-free culture system on recombinant human vitronectin and using StemPro hESC serum-free medium (Life Technologies, CA) supplemented with 20 ng/ml basic fibroblast growth factor (Peprotech, NJ). Cells were passaged mechanically using StemPro EZPassage mechanical stem cell passaging tool (Life Technologies Europe, Bleiswijk, Netherlands). Protocols allowing generation of EC via direct and haemogenic routes are described in the **Supplementary Method**.

***Study population.*** Tissue samples and vessel-wall derived EC were obtained from patients with critical limb ischemia (patients with (*n* = 9) or without (*n* = 10) critical limb ischemia from leftover limb muscle) and premature coronary artery disease (EC derived from the superficial forearm veins, *n* = 8) respectively, following written informed consent, and in accordance with the Declaration of Helsinki. Patient characteristics and protocols for muscle biopsy and isolation of EC are described in the **Supplementary Method** (**Supplementary Tables S5** and **S6**).

Reagents and other detailed methods are described in the **Supplementary Method, Supplementary Tables S1 to S4** and **Supplementary References**.

**SUPPLEMENTARY MATERIAL** **Figure S1**. Schematic representation of genomic localization of both version of *SENCR* and *FLI1.* **Figure S2**. hESC-EC direct differentiation using the H9 and H1 hESC line. **Figure S3**. MACSorting of d7 hESC-EC from direct differentiation. **Figure S4**. Identification of a HEC population existing on d7 of an established hematopoietic differentiation protocol. **Figure S5**. Optimisation of an indirect hESC-EC differentiation protocol. **Figure S6**. Identification of CD326^low^CD56^high^ MP population during direct and indirect hESC-EC differentiation. **Figure S7**. Purification and characterisation of a CD326^low^CD56^high^ MP population in hESC-EC differentiation. **Figure S8**. Expression of *FLI1* during differentiation of hESC into EC and erythroid cells. **Figure S9**. Time course expression of *ETV2, FLI1* and *SENCR* during hESC-EC differentiation and in HUVEC. **Figure S10**. Assessment of toxicity after LV infection using Zombie aqua staining. **Figure S11**. *SENCR* enhances differentiation of CD326^low^CD56^high^ and CD144^+^CD31^+^ hESC-EC. **Figure S12**. *SENCR* expression analysis after VEGF treatment in HUVEC. **Table S1**. Information for specific antibodies used in FACS analysis and sorting. **Table S2**. Information for isotype control antibodies used in FACS analysis and sorting. **Table S3**. Information for probes used in TaqMan-based quantitative real-time PCR assays. **Table S4**. Primer-pairs used for SYBR-Green-based quantitative real-time PCR assays. **Table S5**. Clinical characteristics of patients with or without critical limb ischaemia, from whom leftover limb muscle samples from cardiovascular surgery were analysed. **Table S6**. Clinical characteristics of patients with or without premature coronary artery diseases. **Methods**.
