# Supplementary material for: A Role for the Long Noncoding RNA SENCR in Commitment and Function of Endothelial Cells
Source: Mol Ther. 2016 Apr 5;24(5):978–90. doi: 10.1038/mt.2016.41 (PMC4876031; doi:10.1038/mt.2016.41)
Supplement: SUPPLEMENTAL Material [file mt201641x1.pdf]

SUPPLEMENTAL MATERIAL

Supplementary Methods

Cell Culture Conditions

For direct endothelial cells (EC) differentiation of human embryonic stem cells (hESC), an embyroid body (EB)-based method was employed. HESC were dissociated into single cells using Tryple Select (Life Technologies Europe, Bleiswijk, Netherlands) and resuspended in Stemline II Hematopoietic expansion medium (Sigma-Aldrich, St Louis, USA), supplemented with 10 ng/mL bone morphogenetic protein 4 (BMP4, RD, Minneapolis, USA), 10 ng/mL vascular endothelial growth factor (VEGF) (Peprotech, New Jersey USA), 10 ng/mL Wnt3A (RD, Minneapolis, USA) , 5 ng/mL Activin A (Peprotech, New Jersey, USA) and 10 µmol/L Y-27632 (Millipore, Temecula, USA). Cells were transferred to 96-well round bottom microplates coated with 5% Pluronic F-127 (Sigma-Aldrich, St Louis, USA) as previously described,[1] at a density of 10^4 cells per well and plates were centrifuged at 300 x g for 3 min. A further addition of 16.6µL Stemline II Hematopoietic expansion medium, supplemented with 140 ng/mL BMP4, 210 ng/mL VEGF, 70 ng/mL Wnt3A and 35 ng/mL Activin A, was added to each well on day 2. At day 3, EB were collected from wells, resuspended in endothelial growth medium-2 (EGM-2, Lonza, Basel, Switzerland) supplemented with 50 ng/mL VEGF, and transferred to 6 well cell culture dishes coated with 0.1% gelatine (Sigma-Aldrich, St Louis, USA). The EGM-2 medium used is produced from the addition of the EGM-1 bullet kit (Lonza, Basel, Switzerland) to EGM-2, without VEGF and foetal bovine serum (FBS) supplements. Cells were then cultured until day 7 when they were harvested and analysed.
Indirect hESC-EC differentiation was performed using a similar, EB-based, modified 10 day hematopoietic differentiation protocol (see below). HESC were washed briefly with PBS before the addition of 3mL Stemline II Hematopoietic expansion medium, supplemented with 10 ng/mL BMP4, 10 ng/mL VEGF, 10 ng/mL Wnt3A, 5 ng/mL Activin A and 2 µmol/L Inhibitor VIII (Calbiochem, Massachusetts, USA). Cells were manually cut into colonies using StemPro® EZPassage tools (Life Technologies Europe, Bleiswijk, Netherlands) and transferred to a Corning® 6-well ultra-low attachment plate (Corning, St Lowell, USA) at a 1:3 ratio. A further 1.5mL media supplemented with previously mentioned cytokines, was added to each well. At day 2, cells had formed EB and a further addition of 0.5mL Stemline II Hematopoietic expansion medium and cytokines was made, to give a final concentration of 20 ng/mL BMP4, 30 ng/mL VEGF, 2 µmol/L Inhibitor VIII, 10 ng/mL Wnt3A, 5 ng/mL Activin A, 10 ng/mL fibroblast growth factor-α (FGFα) (Peprotech, New Jersey USA), 20 ng/mL stem cell factor (SCF) (Invitrogen, Paisley, UK) and 0.4 ng/mL β-Estradiol (Sigma-Aldrich, St Louis, USA). On day 3 cells were collected, dispersed, using Tryple Select and resuspended in Stemline II Hematopoietic expansion medium. They were then counted, and seeded at $5x10^5$ cells per well in a standard $25cm^2$ cell culture flask (Corning, St Lowell, USA), in 5mL per well Stemline II Hematopoietic expansion medium containing 20 ng/mL BMP4, 30 ng/mL VEGF, 10 ng/mL FGFα, 30 ng/mL SCF, 0.4 ng/mL β-Estradiol, 10 ng/mL insulin-like growth factor-2 (IGF2) (Peprotech, New Jersey USA), 10 ng/mL thromobopoietin (TPO) (Peprotech, New Jersey USA), 5 ng/mL heparin (RD, Minneapolis, USA), and 50 µmol/L IBMX (Sigma-Aldrich, St Louis, USA). A further 0.5mL media, supplemented with identical cytokines to day 3, was added on day 5, before a complete media change was then performed on day 7. For continuation to of haematopoietic differentiation, a final addition of 0.5mL media and cytokines was made on day 9, before cells were harvested for analysis on day 10. For differentiation to EC, media was removed, and exchanged for EGM-2 medium (prepared as previously described) with 50 ng/mL VEGF.
Erythroid differentiation from H1 hESC lines was performed according to a patented method (WO/2014/013255).[2] Briefly, we used a multi-step, feeder-free protocol, to initiate differentiation EB were formed in Stemline II medium with BMP4, VEGF, Wnt3a and Activin A. In a second step haematopoietic differentiation was induced by adding FGFα, SCF, IGF2, TPO, heparin to the EB factors. After 10 days, haematopoietic progenitors were harvested and replaced into fresh Stemline II Hematopoietic expansion medium supplemented with BMP4, SCF, Flt3 ligand (Peprotech, New Jersey, USA), interleukin-3 (Peprotech, New Jersey, USA), interleukin-11 (Peprotech, New Jersey, USA), EPO (Roche, New South Wales, Australia) to direct differentiation along the erythroid lineage

and to support extensive proliferation. After 17 days cells were transferred into Stemline II Hematopoietic expansion medium containing a more specifically erythroid cocktail that included insulin (Sigma-Aldrich, St Louis, USA), transferrin (Sigma-Aldrich, St Louis, USA), SCF, insulin-like growth factor-1 (Peprotech, New Jersey USA), IL3, IL11 and EPO for 7 days. In a final maturation step of 7 days (day 24) cells were transferred into Iscove's Modified Dulbecco's Medium (Life Technologies Europe, Bleiswijk, Netherlands) with insulin, transferrin and BSA (Life Technologies Europe, Bleiswijk, Netherlands), supplemented with EPO. Cells were harvested for analysis on day 10, 17 and 24.

Human umbilical vein endothelial cells (HUVEC) were purchased from Lonza (Verviers, Belgium) and cultured in Media 200 (Life Technologies, California, USA) supplemented with 1 µg/mL hydrocortisone (Life Technologies, California, USA), 10 ng/mL human epidermal growth factor (Life Technologies, California, USA), 3 ng/mL basic fibroblast growth factor (Life Technologies, California, USA), 10 µg/mL heparin (Life Technologies, California, USA), 20% heat-inactivated FBS, 50 µg/mL penicillin, 50 µg/mL streptomycin, 2 mmol/L L-Glutamate, and 1mmol/L sodium pyruvate (all from : Invitrogen, Paisley, UK).

HEK293T cells (ATCC, Teddington, UK) were maintained in Dulbecco's modified Eagle's medium (DMEM; Invitrogen) supplemented with 10% heat-inactivated FBS, 50 µg/mL penicillin, 50 µg/mL streptomycin, 2 mmol/L L-Glutamate, and 1mmol/L sodium pyruvate (All from : Invitrogen, Paisley, UK).

Magnetic Activated Cell Sorting (MACS)

Magnetic activated cell sorting of $CD144^+$ cells was performed using commercially available kits from Miltenyi Biotec, following the recommended protocol (Miltenyi Biotec, Bergisch Gladbach, Germany). Flow cytometry was performed to confirm efficient purification of $CD144^+$ cells. Sorted cells were then plated onto gelatine coated 6-well tissue culture plates at 10^5 cells per well and cultured for a further 7 days in EGM-2 media with FBS component supplemented with 50 ng/mL VEGF.

Lentiviral Vectors Production and Cell Infection

Lentiviral vectors were produced by triple transient transfection of HEK293T cells with a packaging plasmid (pCMVΔ8.74), a plasmid encoding the envelope of vesicular stomatitis virus (VSVg) (pMDG) (Plasmid Factory, Bielefeld, Germany) and pLNT/*SFFV*-MCS plasmid employing polyethylenimine (PEI; Sigma-Aldrich, St Louis, USA) as previously described.[3] Lentiviral titres were ascertained by TaqMan quantitative real-time PCR (qRT-PCR) using the following primer/probe sequences: forward, 5'-TGTGTGCCCGTCTGTTGTGT-3'; reverse, 5'-GAGTCCTGCGTCGAGAGAGC-3'; probe, 5'-(FAM)-CAGTGGCGCCCGAACAGGGA-(TAMRA)-3.[4] All lentivirus batches used for experiments had comparable titers ranging from 10^8 to 10^9 transducing functional U/mL. Virus suspensions were stored at -80°C until use and were briefly centrifuged and kept on ice immediately before use.

Plasmid pcDNA3.1-*SENCR* was cloned between NheI and XbaI restrictions sites. The following primers were used to flank *SENCR* insert with Mlul1 and BamHI restriction sites : forward 5'-ATGCGGATCCTTCTTCACTTTTCCTTAGGGGTGT-3' and Reverse 5'-TCTAGAACGCGTCTCTAAAGTGCTAGGAGGAGGGT-3'. *SENCR* insert was then cloned into the pLNT/*SFFV*-MCS plasmid (kind gift from Adrian J. Thrasher, London, UK) to obtain the construct pLNT/*SFFV-SENCR*.

For hESC infection, in pluripotent state, adherent pluripotent H9 hESC were transduced with a multiplicity of infection (MOI) of 10. Cells were incubated in pluripotent maintenance media containing LV particles and 4 µg/mL polybrene (Sigma-Aldrich, Milwaukee, USA) for 18 h at 37°C in a humidified atmosphere containing 5% CO2. LV particles were removed, and media were replaced with fresh pluripotent maintenance media for an additional 72 h. Pluripotent maintenance media was changed every 24 h.

For LV infection during EB formation, 7 x 10^5 H9 hESC in suspension were transduced with a MOI of 10. Cells were incubated in Stemline II Hematopoietic Expansion Medium for 30 min at 37°C in a

humidified atmosphere containing 5% CO2. Then cells were subjected to direct endothelial differentiation as described above.
For HUVEC, 8 x 10^4 cells were transduced with MOI of 10. Cells were incubated in endothelial media containing LV particles for 18 h at 37°C in a humidified atmosphere containing 5% CO2. LV particles were removed, and media were replaced with fresh endothelial media for an additional 72 h.

Fluorescent activated cell sorting (FACS) analysis and sorting

Cells were harvested by enzymatic dissociation and washed in PBS. Subsequently, cells were incubated for 1 h at 4°C with the specific antibodies (BD Biosciences, San Diego, USA) (Table S 1). Corresponding isotype controls (BD Biosciences, San Diego, USA) (Table S2) were used to calibrate the settings on the flow cytometer. The samples were then analysed with a flow cytometer (BD Canto II, San Diego, USA). Singly-stained compensation controls and fluorescence minus one (FMO) controls were performed to ensure correct compensation of the cytometer, and antibodies were titrated to ensure appropriate amounts were used. The data were collected and analysed with FlowJo software (Ashland, OR, USA).
FACS sorting was performed using APC mouse anti-human CD326 and PE mouse anti human-CD56, and a FACSAria I or Aria III cell sorter (BD Biosciences, San Diego, USA).

HUVEC proliferation assay

The cultured HUVEC were plated in 0.1% gelatine coated 6-well (8.10^4) or 94-well (10^4) plates. At 70-80% confluence the cells were starved with starvation media consisting of DMEM supplemented with 2% heat-inactivated FBS, 50 µg/mL penicillin, 50 µg/mL streptomycin, 2 mmol/L L-Glutamate and 1mmol/L sodium pyruvate for 48 h then treated or not with 50 ng/mL VEGF in starvation media for 6, 12 and 24 h. For RNA extraction, cells were harvested at indicated time and RNA extracted as indicated below. For the proliferation assay, bromodeoxyuridine (BrdU) reagent (Millipore, Molsheim, France) was added directly to cell culture during cell proliferation and incubated for 12 and 24 h. The cells were fixed, permeabilized and then the DNA was denatured using a fixative solution. The anti-BrdU monoclonal antibody was added and incubated at room temperature for 1 h. Cells were washed and incubated with Horseradish Peroxidase-Conjugated Goat anti-Mouse antibody at room temperature for 30 min. Cells were washed, TMB Peroxidase Substrate was added and cells were incubated at room temperature in the dark for 30 min. The reaction was ended by the addition of Stop Solution. Optic absorption at 450 nm was measured to detect BrdU, using a plate reader. The proliferation was expressed as optical density (OD) mean values in the presence of BrdU.

SiRNA Transfection

To knockdown SENCR in HUVEC, a siPOOL characterized by 30 different siRNA targeting the same gene were utilized (Sitoolsbiotech, Planegg, Germany). The standard negative control siPOOL correspond to a pool of 30 siRNA which do not interact with human gene (Sitoolsbiotech, Planegg, Germany). HUVEC (8 × 10^4/well) were plated in 6-well plates coated with gelatine during 24 h. Cells were then transfected with 10 nmol/L siRNA control and 10 nmol/L siRNA against *SENCR* for 48 h using Lipofectamine RNAiMax transfection reagent (Life technologies, California, USA) according to the manufacturer's protocol. Following the transfection, HUVEC were then harvested for experiments as indicated.

Cell Viability

A Live/Dead assay (ZombieAqua Flexible, Biolegend, California, USA) was utilized to investigate the effect of LV transduction on cell viability. The cells were infected as described above. After the respective time of culture, cells were harvested, suspended in FACS buffer (1% KOSR, 5 mmol/L Ethylenediaminetetraacetic acid (EDTA)) and then stained with 1µL of ZombieAqua Flexible for 15 min. The samples were then analysed with a flow cytometer (BD Canto II, San Diego, USA). The unstained groups were used to calibrate the settings on the flow cytometer. The data were collected and analysed with FlowJo software.

Wound Healing Assay

HUVEC were transfected or infected with siPool and LV particles, respectively, and grew into confluent monolayers overnight. Cells were then subjected to a starvation in DMEM supplemented with 2% heat-inactivated FBS, 50 µg/mL penicillin, 50 µg/mL streptomycin, 2 mmol/L L-Glutamate and 1 mmol/L sodium pyruvate. Wound healing was generated with a sterile 200µL pipette tip. Cell migration towards the wound was monitored at 0 h and 12 h using a light microscope. Quantification of migrated distance was done using ImageJ.

Cell cycle analysis

Cell cycle analysis was performed using propidium iodide staining and flow cytometry. HUVEC transfected with SiPool and transduced by LV particles were dissociated at 48 h and 72 h, respectively. After fixing the cells with ice-cold 70% ethanol for 15 min, the cells were centrifuged and stained with propidium iodide (Sigma-Aldrich, Gillingham, UK) for 30 min. The samples were then analysed with a flow cytometer (BD Canto II, San Diego, USA). The unstained and stained cell groups were used to calibrate the settings on the flow cytometer. The data were collected and analysed with FlowJo software.

***In vitro* angiogenesis**

Testing for HUVEC tube formation was performed in a 96-well plate containing 50µL Matrigel (in vitro angiogenesis assay ECM625, Millipore, USA) according to the manufacturer's protocol. Briefly, 50µL Matrigel-solution were added to one well of a 96-well plate and allowed to solidify and polymerize at 37°C and 5% CO2 for 1 h. Transfected and infected HUVEC were then dissociated, centrifuged at 1200 RPM for 5 min and resuspended in endothelial media. Finally, 100µL of endothelial media containing 10^4 cells were layered on top of the Matrigel. After 12 h incubation at 37°C and 5% CO2, tube formation was inspected using a light microscope. Tube-like formations were evaluated in 15 pictures per condition (magnification 10×) and the number of cells, number of branches, average length of the capillary branches, number of branches to the length of capillaries ratio and the pattern were assessed using angiogenesis analyser for Image J.[5]

TaqMan Q-RT PCR Analysis of Mature miRNAs, mRNAs and lncRNA

Total cellular RNA were isolated using miRNAeasy Mini Kit (Qiagen, Crawley, U.K., http://www.qiagen.com). Total RNA was reverse transcribed using specific miRNA primers provided with TaqMan miRNA assays or reverse transcribed using random primers for mRNA and lncRNA expression analysis. Human miRNAs and mRNAs were analysed using TaqMan assays (Life Technologies, Paisley, UK) (Table S3). Ubiquitin C (*UBC*), glyceraldehydes 3-phosphate dehydrogenase (*GAPDH*) and *RNU40* have been used for normalization (Life Technologies, Paisley, UK) (Table S3). Human lncRNA were analysed with SYBR Green PCR Master Mix (Life Technologies, Paisley, UK) using specific forward and reverse primer for *SENCR V1, SENCR V2* and *MALAT1* (Eurofins Genomics, Ebersberg). *UBC* and *GAPDH* have been used for normalization (Eurofins Genomics, Ebersberg) (Table S4). Quantitative PCR reactions were performed in technical duplicates with the 7500 Fast Real-time PCR System (Life Technologies, Paisley, UK). Quantitative expression was calculated using the $2^{-(\Delta\Delta CT)}$ (RQ) or $2^{-(\Delta CT)}$ method, where ΔΔCt= ΔCt treated cells-ΔCt control cells and ΔCt= test gene CT – housekeeping gene CT.

RNA fractionation

RNA fractionation was performed according to the manufacturer's instructions (Ambion, Paris Kit, Life Technologies, California, USA). Briefly, HUVEC and hESC-EC d7 were dissociated then first homogenized in ice-cold Cell Fractionation Buffer and incubated 5 min at 4C. After centrifugation, pellets containing intact nuclei were resuspended in Cell Disruption Buffer. The supernatant contains all of the cytoplasmic components. For RNA isolation, each part of the cell lysate (nuclear and cytoplasmic) were mixed with an equal volume of Lysis/Binding Solution. Total RNA was then purified from the mixture using a RNA binding glass fiber filter. After three rapid washing steps,

concentrated RNA was eluted. The equal volumes of nuclear and cytoplasmic RNA, corresponding to equivalent numbers of cells were used for the first strand cDNA synthesis. The abundance of *SENCR* and *NEAT1* transcript in nuclear and cytoplasmic fraction was analysed by qRT-PCR and normalized using *UBC* using the $2^{-(\Delta CT)}$ method.

RNA fluorescence in situ hybridization (FISH)

RNA FISH assay was performed according to the protocol of Affimetrix (https://www.panomics.com/downloads/UM18801_QGViewRNA_ISH_CellAssay_RevA_110525.pdf). Custom probe oligonucleotide specific for *SENCR* long isoforms was designed and synthesized by Affymetrix as Type 1. Probe specific for human Type 6 *UBC* and Type 6 *SNORD3A* were used as cytoplasmic and nuclear controls, respectively. Cell nuclei were visualized with DAPI. Images were acquired on a confocal microscope (Zeiss 510, Germany).

Microarray analyses

HUVEC were transfected with control siRNA or siRNA against *SENCR* for 48 h and total RNA was extracted as described above. In vitro transcription was performed using the Ambion Illumina TotalPrep RNA Amplification Kit (Life Technologies, Paisley, UK). The microarray was performed using the Illumina Human v2.0 Expression BeadChip Kit (Illumina, Braintree, UK) and data were generated using GenomeStudio (Illumina, Braintree, UK) (n=2 per group). To assess the statistical significance of pairwise intergroup differences, LIMMA R-software library was used. Significance was assessed using the false discovery rate (FDR) multiple testing correction method with a FDR cut-off of 5%. A secondary analysis was conducted using linear models for microarray data, and the significance cut-off was a FDR adjusted P value of 0.05). Data were analysed using the IPA (Ingenuity Systems, www.ingenuity.com).

TLDA

For stem cells adhesion gene PCR arrays, custom TaqMan Low Density Array cards (TLDA) were used according to manufacturer's instructions on an Applied Biosystems 7500 Fast Real-time PCR System (Life Technologies, Paisley, UK).

Critical Limb Ischemia Samples

Anonymized leftover limb muscle samples (adjacent to the surgical incision) were obtained from patients receiving limb amputation for critical limb ischemia (CLI) and from subjects with no known peripheral vascular disease who were undergoing saphenous vein harvesting in preparation for elective coronary artery bypass grafting (non-CLI control) (Table S5). Patient characteristics are reported in the online-only Data Supplement (Table S5). All muscle samples were collected in RNALater® RNA stabilization solution (Life Technologies, Paisley, UK), incubated overnight at 4°C and stored at -80°C, as per the manufacturer's protocol. Total RNA was extracted from critical limb ischemic tissues from muscle biopsy using TRIzol solubilization method and RNA concentration was measured with the NanoDrop spectrophotometer. For reverse transcription, cDNA was synthesised from 500 ng of total RNA using the QuantiTect RT kit (Qiagen, Manchester, UK), as per the manufacturer's instructions, and included a step to remove genomic DNA. All cDNA was stored at -20°C. Quantitative PCR was performed using Power SYBR (Life Technologies, Paisley, UK) with a LightCycler 480 (Roche, St Albans, UK), according to the manufacturer's instructions. Data were normalised to *18s* as an endogenous housekeeping gene, and relative expression was calculated using the $2^{-(\Delta\Delta CT)}$ method.

Isolation of endothelial cells from patients with premature coronary artery disease

Patients included in the study had either a previous myocardial infarct or evidence of coronary artery disease on coronary angiography and were under 50 years of age at the time of the study (Table S6). Family history of premature coronary artery disease was defined as any first degree relative with

coronary artery disease (i.e., angina, previous coronary artery bypass grafting or percutaneous coronary intervention), myocardial infarction or sudden cardiac death without obvious cause diagnosed at age <55 years for men or <65 years for women. A control group of healthy age- and sex-matched subjects (n=8) was recruited from the Clinical Research Imaging Centre at the Royal Infirmary of Edinburgh using the criteria of no evidence of significant coronary artery disease following CT coronary angiography (CTCA). Exclusion criteria included: diabetes mellitus, current cigarette smokers, systolic blood pressure >190 mmHg or <100 mmHg, renal dysfunction (serum creatinine >200 µmol/L or estimated glomerular filtration rate <30 mL/min), hepatic dysfunction, history of blood dyscrasia, blood donation within the last 3 months, inability to provide informed consent, pregnancy, anti-coagulant medication or an inter-current illness (<6 weeks). The patient group had additional exclusion criteria including acute coronary syndrome (<3 months), symptoms of heart failure, moderate or severe left ventricular dysfunction, or significant left main stem disease (>50%) or three-vessel coronary artery disease and had not been revascularised (Table S4). An adapted protocol was used to isolate local vessel wall endothelial cells.[6] Under local anaesthetic (1% lidocaine), an 18-gauge venous cannula was inserted into a superficial forearm vein and a J-shaped guidewire (0.018 inch diameter) passed and gently manipulated to harvest endothelial cells. The wire was transferred to Endothelial Cell Growth Medium-2 (EGM™-2; Lonza, UK), and inserted into sterile Masterflex Tygon® tubing (Cole Parmer Instrument Company Limited, UK). Medium was syringed through the tubing to detach cells, which were collected by centrifugation and seeded into BD BioCoat Collagen 1 coated 6 well plates (Scientific Laboratory Supplies Ltd., UK). Cells were incubated under standard culture conditions in EGM™-2 medium with the addition of 10% defined foetal bovine serum Thermo Scientific Hyclone (Fisher Scientific, UK). Medium was replenished every 2-3 days until the first passage.

Total cellular RNAs from cells was isolated using miRNAeasy Mini Kit from cells at passage 3-4 (Qiagen, Crawley, U.K., http://www.qiagen.com) as described above. Total RNA was reverse transcribed as described above using specific random primers for lncRNA expression analysis. *SENCR* expression was analysed and *UBC* used for normalization (Supplemental Table 4). Quantitative PCR reactions were performed in technical duplicates with the 7500 Fast Real-time PCR System (Applied Biosystems) and relative expression was calculated using the $2^{-(\Delta\Delta CT)}$ method.

Statistical analysis

ANOVA with Tukey's HSD post-hoc comparisons, Student's one sample t-tests and Student's two sample t-tests were used to determine statistical significance of the means (± standard deviation) and graphs were plotted using Graph-Pad Prism 5.0. Statistical significance was assumed at $p < 0.05$.

Chromosome 11

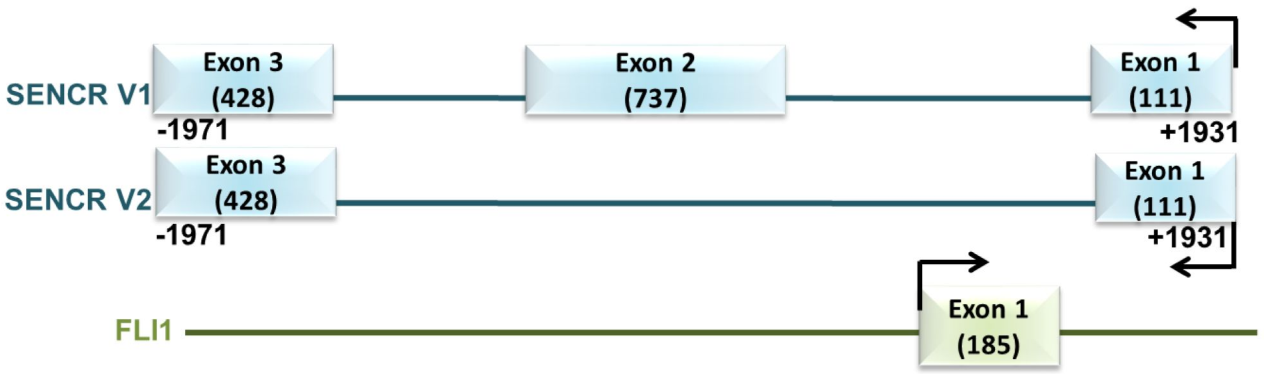

Figure S1. Schematic representation of genomic localization of both version of *SENCR* and *FLI1.*

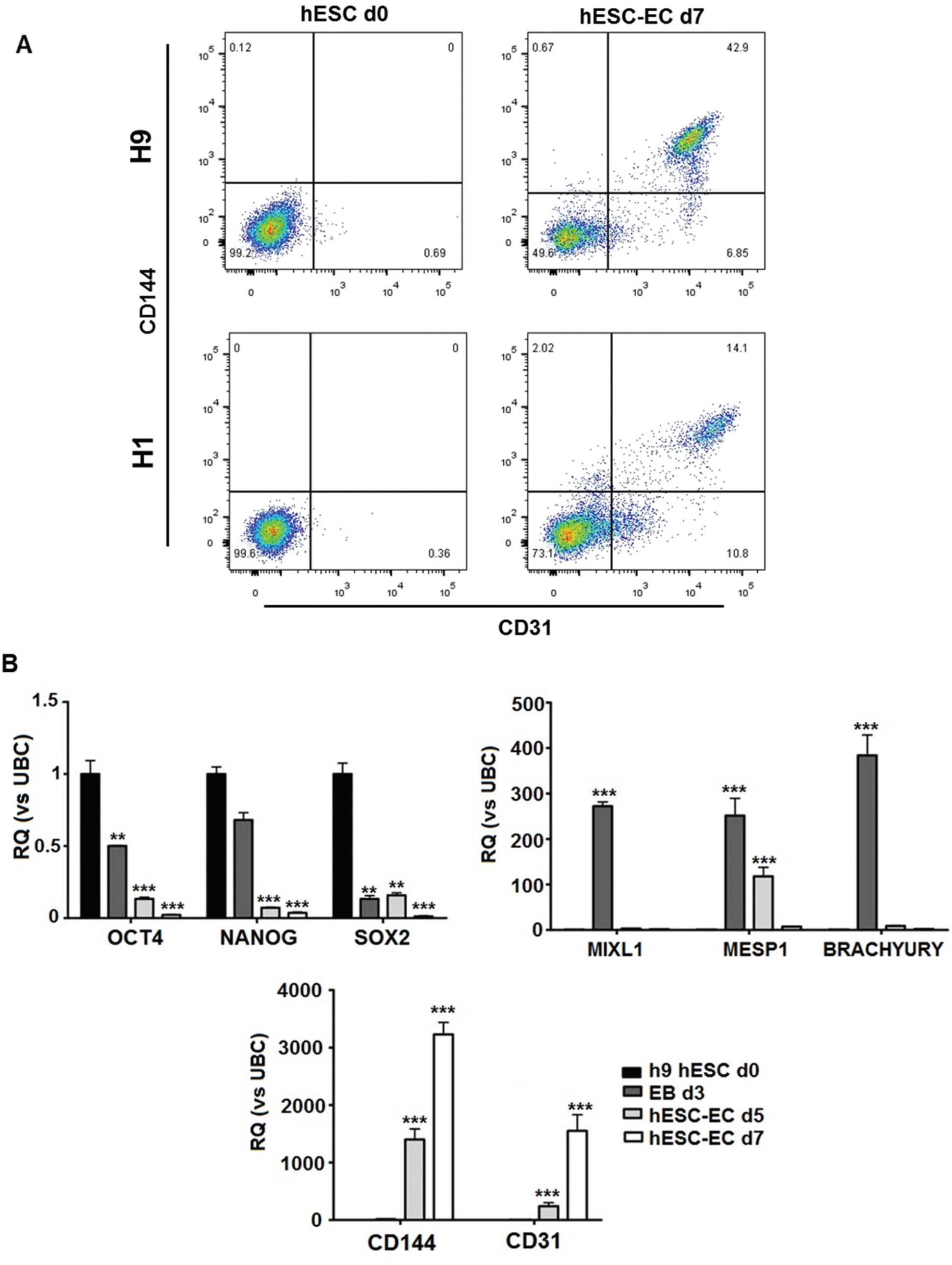

Figure S2. hESC-EC direct differentiation using the H9 and H1 hESC line. A. Representative dot plots for CD144 and CD31 staining on pluripotent d0 H9 (top) and H1 (bottom) hESC *versus* d7 hESC-EC. B. QRT-PCR analysis of pluripotent (left top), mesodermal (right top) and endothelial-associated (bottom) genes in pluripotent d0 H1 hESC, d3 EB and d5 and d7 hESC-EC. Histogram shows RQ calculated using the $2^{-\Delta\Delta cT}$ method and error bars are RQ_{max}. n=3, repeated measures ANOVA, Tukey's t-test, *=P<0.05, **=P<0.01, ***=P<0.001.

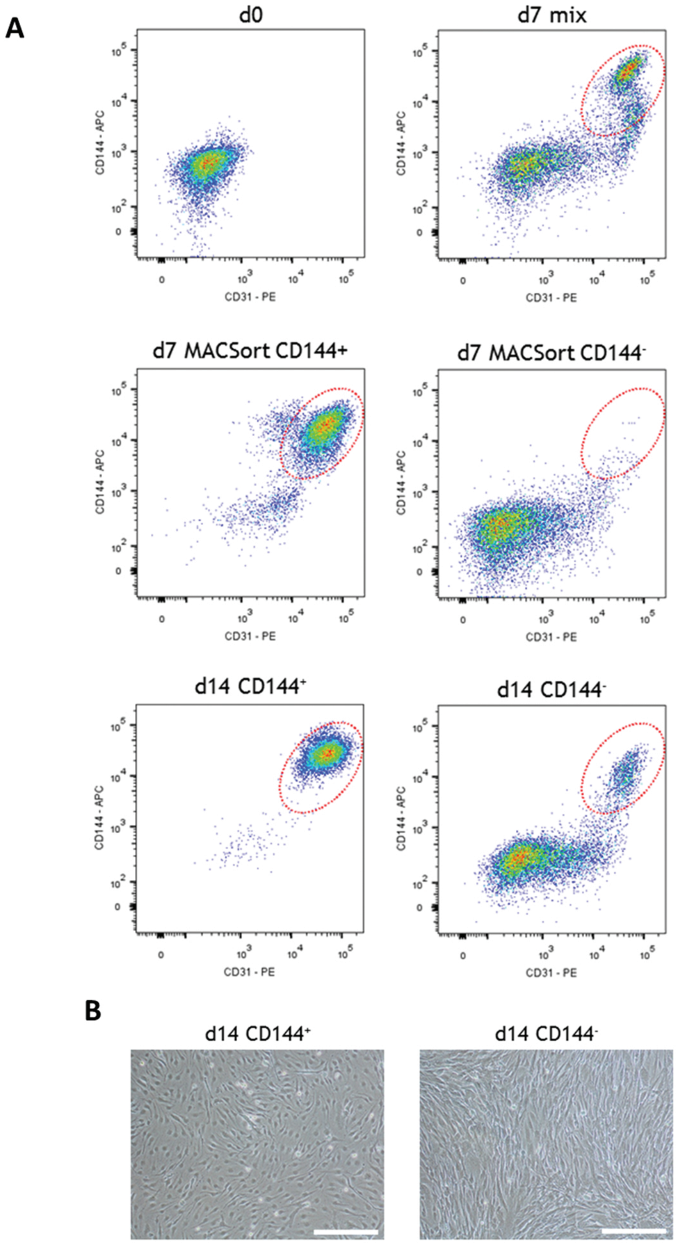

Figure S3. MACSorting of d7 hESC-EC from direct differentiation. *A. Staining of d0, d7 mixed hESC-EC, d7 MACSorted CD144+, d7 MACSorted CD144-, d14 cultured CD144⁺ and d14 cultured CD144- cells with APC-conjugated anti-CD144 and PE-conjugated anti-CD31. d7 cells were MACSorted, and both CD144⁺ and CD144⁻ plated out onto gelatin at a density of 10^5 cells per well in a 6-well cell culture dish and cultured for a further 7 days to obtain d14 CD144⁺ and d14 CD144⁻ cells. Representative dot plot for each group shown, red dotted line highlights CD144⁺CD31⁺ population. B. Morphological analysis of d14 CD144⁺ and d14 CD144⁻ hESC-EC. Images are representative for sample group, taken at 10x magnification, scale bar represents 100µm.*

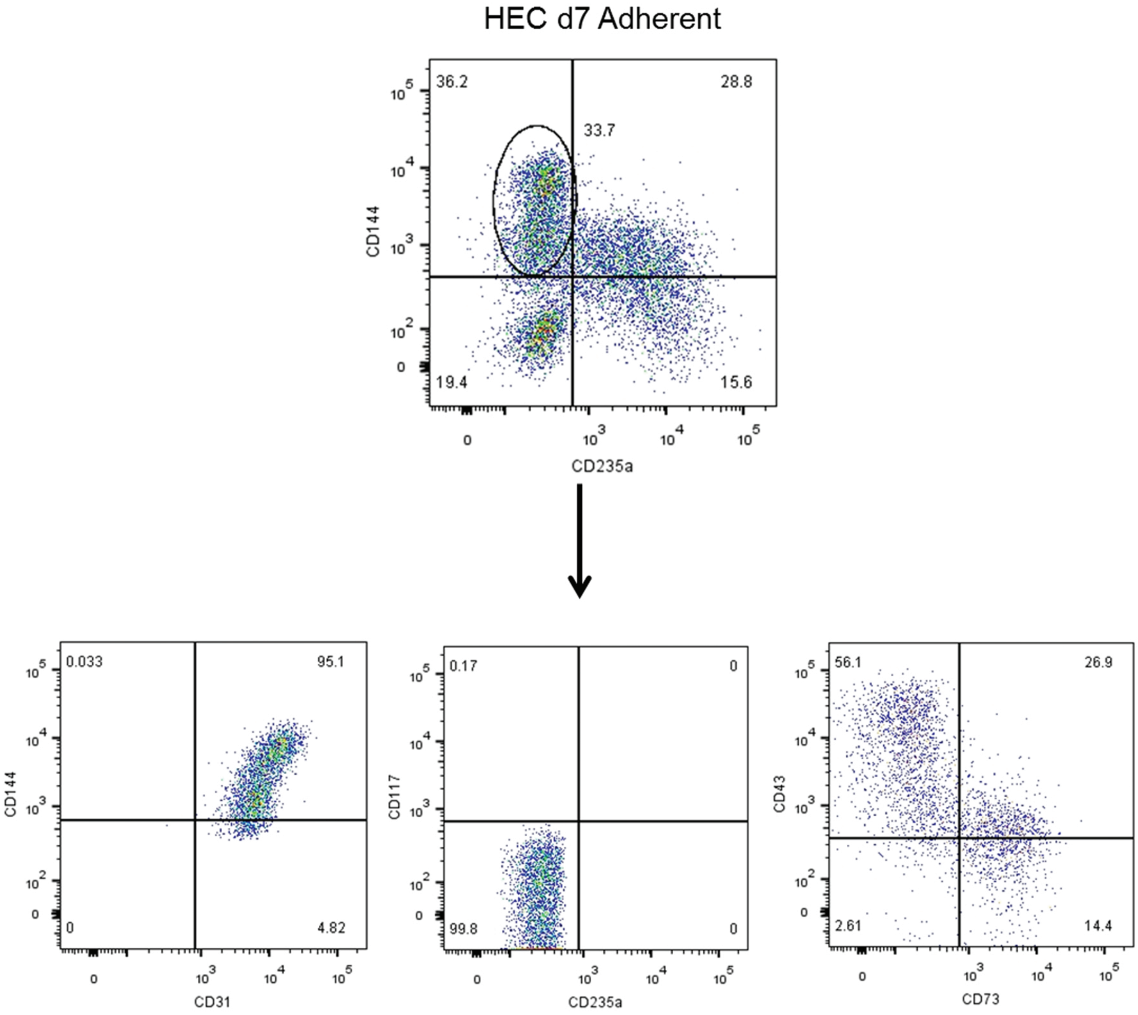

Figure S4. Identification of a HEC population existing on d7 of an established hematopoietic differentiation protocol. *Optimisation of hESC-EC culture from d7 HEC during an established hematopoietic differentiation protocol. 6-colour flow cytometric analysis was employed to profile HEC existing during haematopoietic differentiation of hESC. Cells were stained with APC-conjugated anti-CD63, PE-conjugated anit-CD144, FITC-conjugated anti-CD31, PE-Cy7-conjugated anti-CD43, PerCP-Cy5.5-conjugated anti-CD117 and BV450-conjugated anti-CD235a. Top panel shows heterogeneous cell staining for CD144 and CD235a, circular gate shows CD235a$^-$CD144$^+$ cells. Lower panel shows additional marker profile of the CD235a$^-$CD144$^+$ cells, used as a basic HEC surface marker profile. Dot plots show representative images.*

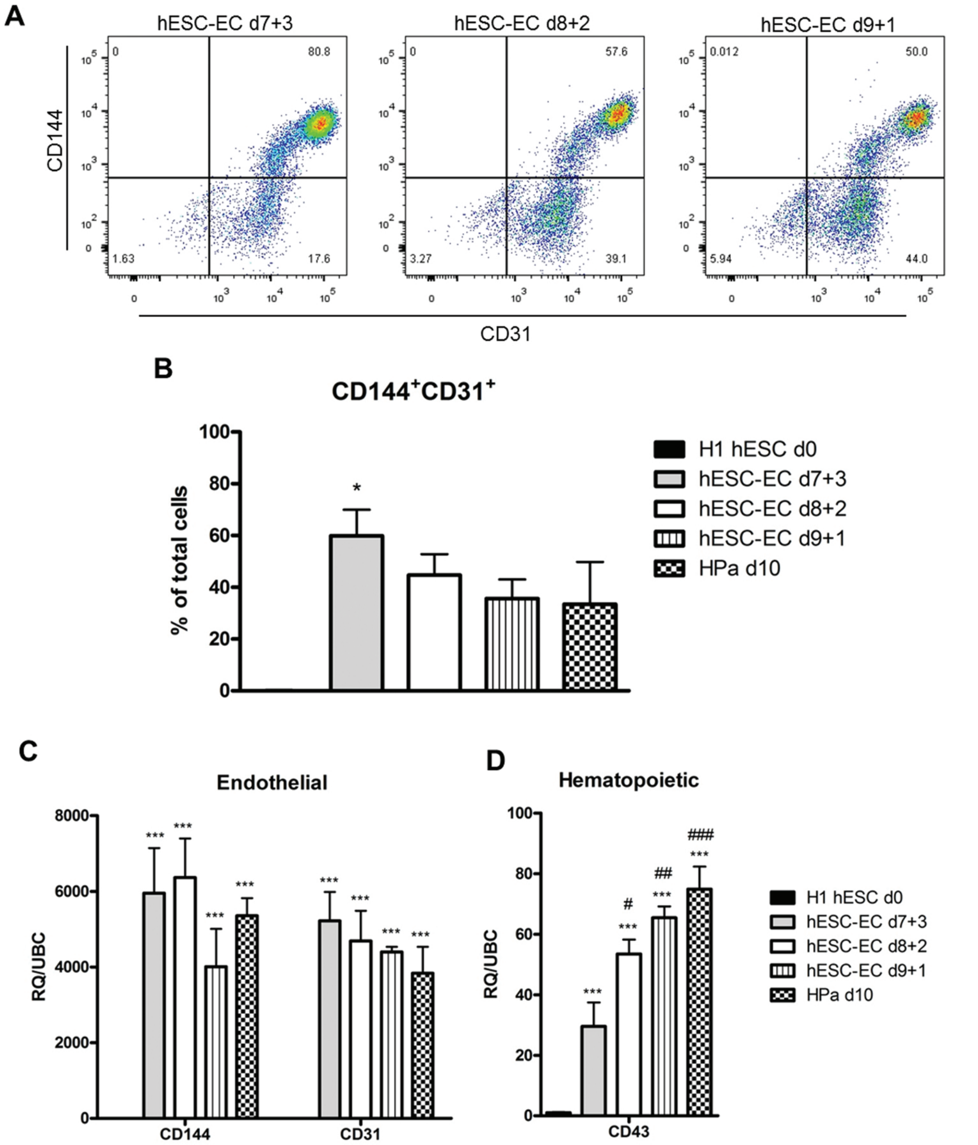

Figure S5. Optimisation of an indirect hESC-EC differentiation protocol. *A. Flow cytometry analysis of $CD144^{+}CD31^{+}$ cells in HEC cultured for 3 days (d7+3 hESC-EC), 2 days (d8+2 hESC-EC) or 1 day (d9+1 hESC-EC) in endothelial supportive conditions. Representative dot plots are shown for each group, stained with APC-conjugated anti-CD144 and PE-conjugated anti-CD31. B. Flow cytometry data plotted as a histogram showing the percentage of total cells positive for CD144 and CD31. n=3, Error bars S.E.M. C-D. QRT-PCR analysis of endothelial (C) and hematopoietic (D) genes in samples from indirect hESC-EC differentiation optimisation experiments, including d7+3, d8+2 and d9+1 hESC-EC. n=3. Repeated measures ANOVA, Tukey's t-test,*P<0.05, **P<0.01, ***P<0.001 compared to H1 d0 hESC; #P<0.05, ##P<0.01, ###P<0.001 compared to d7+3 hESC-EC.*

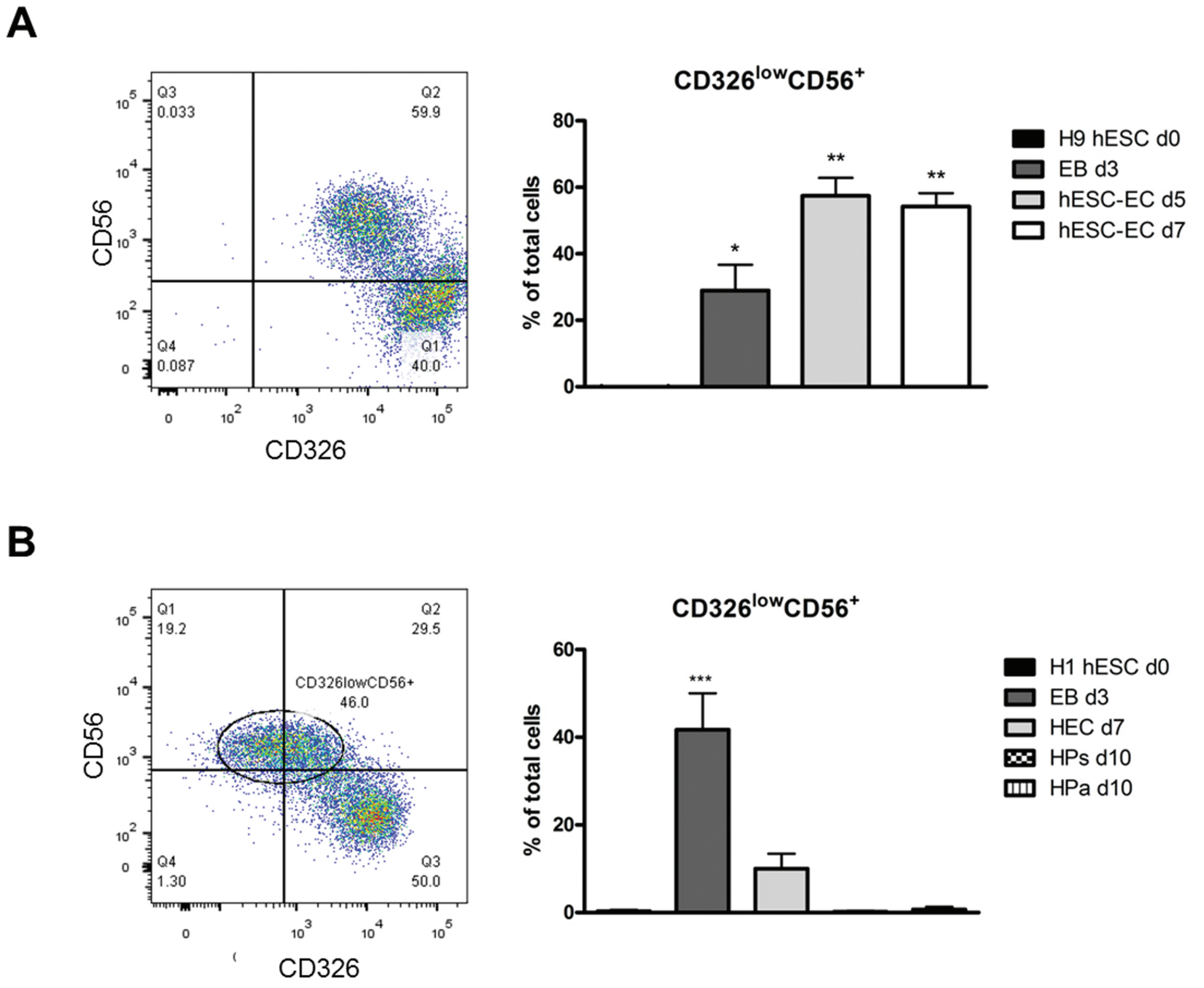

Figure S6. Identification of CD326^{low}CD56^{high} MP population during direct and indirect hESC-EC differentiation. *A-B. Flow cytometric analysis of cells during direct (A) and indirect (B) EC differentiation using APC-conjugated anti-CD326 and PE-conjugated anti-CD56. Dot plot (left) shows representative staining in d3 EB. Histogram (right) shows the percentage of total cells with the CD326^{low}CD56^{high} profile during hESC-EC differentiation. n=3, repeated measures ANOVA, Tukey's t-test, *P<0.05, **P<0.01, ***P<0.001*

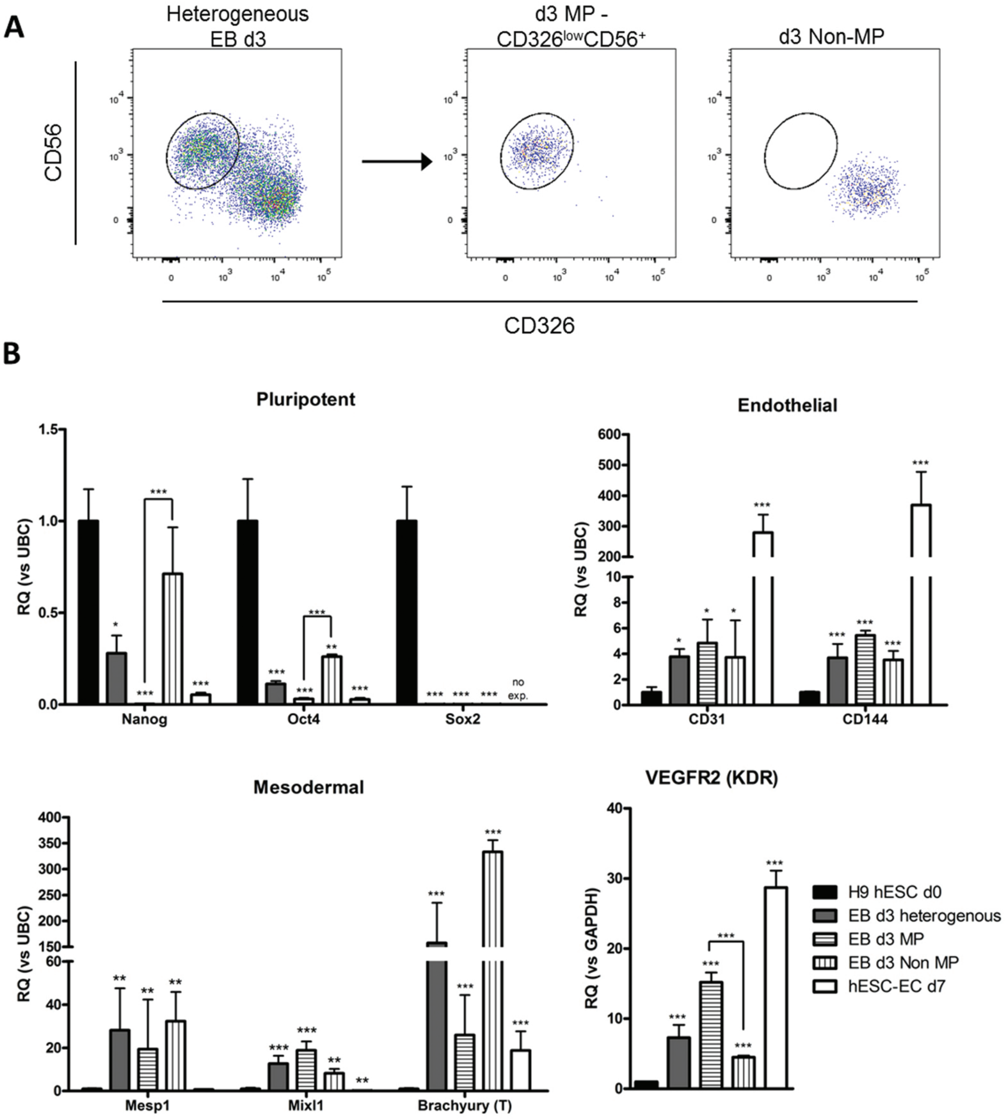

Figure S7. Purification and characterisation of a CD326^{low}CD56^{high} MP population in hESC-EC differentiation. *A. FACSorting of CD326^{low}CD56^{high} MP cells from d3 EB during direct hESC-EC differentiation. Dot plots show representative samples from pre-sort heterogeneous samples and post-sort purified CD326^{low}CD56^{high} MP cells and the 'Negative' fraction from the sort (non-MP). B.* *qRT-PCR analysis of gene expression of RNA collected from d0 H9 hESC, heterogeneous d3 EB, d3 CD326^{low}CD56^{high} MP, d3 non-MP and d7 hESC-EC. Pluripotency, mesodermal and endothelial-associated genes were profiled, as well as VEGFR2 (KDR). Histograms show RQ calculated relative to d0 pluripotent control, error bars RQ$_{max}$. Repeated measures ANOVA, Tukey's t-test, *P<0.05, **P<0.01, ***P<0.001, compared to d0 pluripotent control, unless specified.*

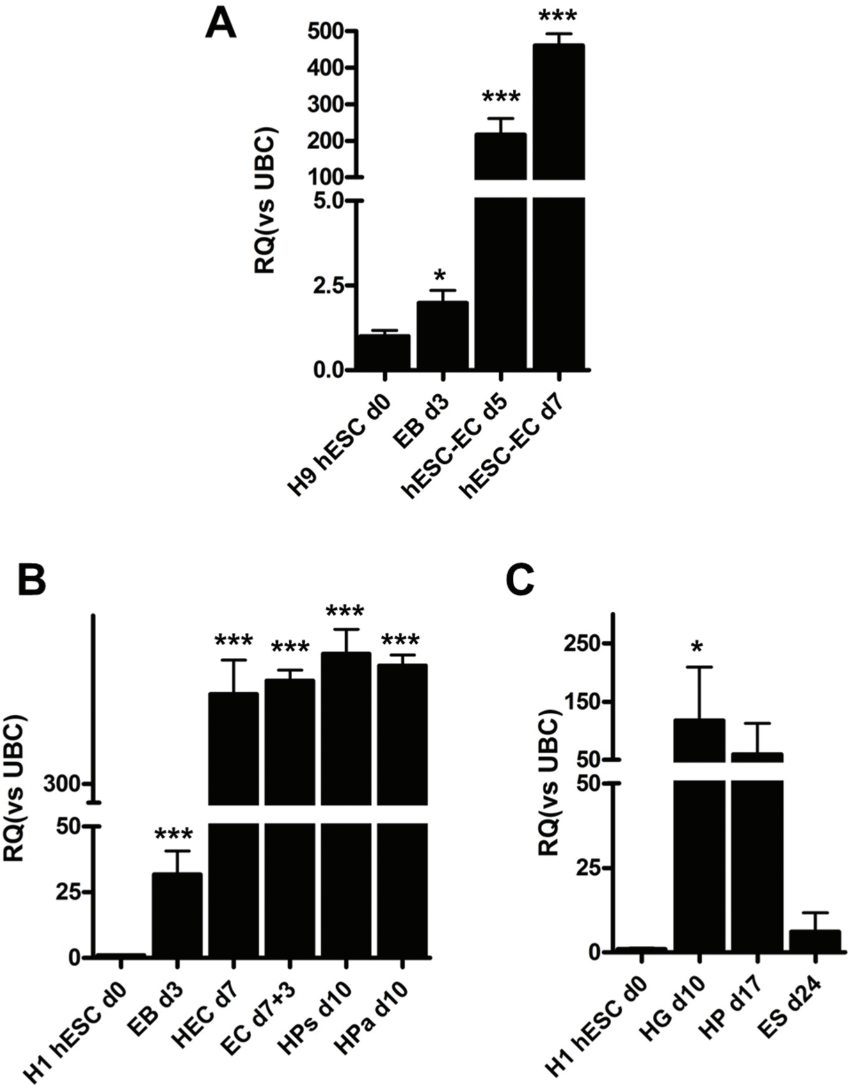

Figure S8. Expression of *FLI1* during differentiation of hESC into EC and erythroid cells. *Quantification of FLI1 gene expression and Pearson's correlation calculated as measure of the degree of linear dependence between between FLI and SENCR during: A. Direct differentiation of hESC into EC versus H9 hESC compared to EB at d3 and mixed population of hESC-EC at d5-7. Pearson correlation, r=0.9992, P<0.001; B. Indirect differentiation of hESC into EC versus H1 hESC compared to d3 EB, d7 HEC, d7+3 hESC-EC, d10 HPs/a. Pearson correlation, r=0.9466, P<0.01; C. Erythroid differentiation versus H1 hESC compared to haemangioblast at day 10 (HG d10), d17 HP, d24 erythroid cells (ES d24). Pearson correlation r=0.9656, P<0.05. Histograms show RQ calculated relative to d0 pluripotent control, error bars RQ_{max}. n=3, Student's t test, *P<0.05, **P<0.01, ***P<0.001 compared to d0.*

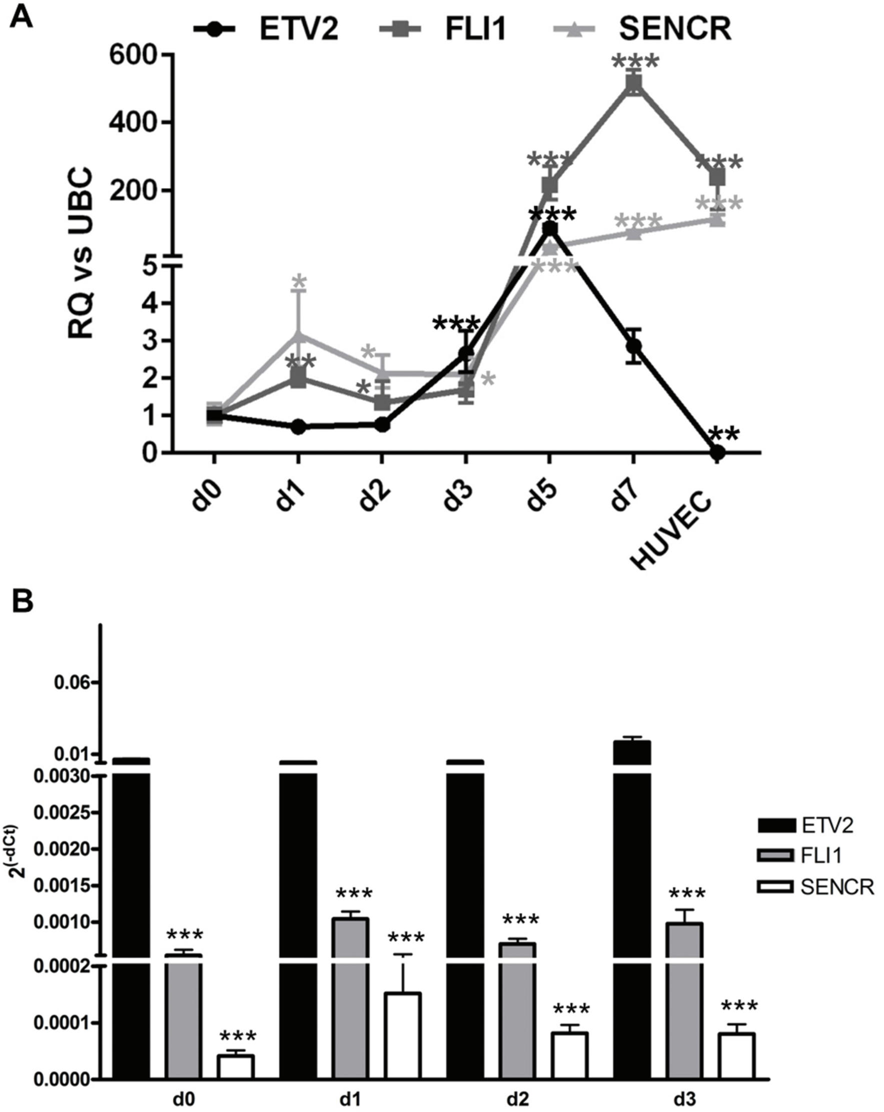

Figure S9. Time course expression of *ETV2, FLI1* and *SENCR* during hESC-EC differentiation and in HUVEC. *A. Time course expression of ETV2, FLI1, SENCR measured by qRT-PCR versus d0 H9 hESC compared to EB at d0, d1, d2, d3; hESC-EC at d5, d7; and HUVEC. Graphs show RQ calculated relative to d0 pluripotent control, error bars RQ_{max}. B. Quantification of ETV2, FLI1, SENCR expression represented by the 2^{-dct} in H9 hESC (d0) and EB at day 1 (d1), 2 (d2) and 3 (d3) demonstrating that ETV2 was the most expressed transcript compared to FLI1 and SENCR. Histogram shows 2^{-dCt} calculated relative to d0 pluripotent control, error bars S.E.M. Student's t test, *P<0.05, **P<0.01, ***P<0.001 compared to d0 for A (black stars=ETV2, dark grey stars=FLI1, light grey stars=SENCR) and compared to ETV2 expression for B.*

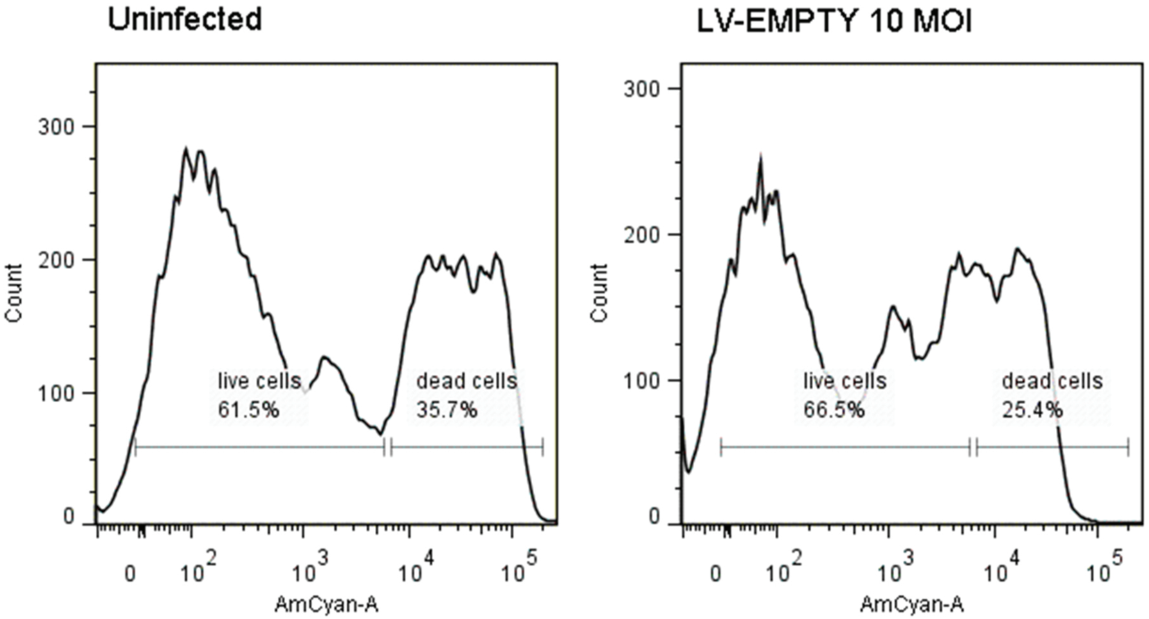

Figure S10. Assessment of toxicity after LV infection using Zombie aqua staining. Three days following infection, EB were dissociated and stained with Zombie Aqua™ and then analysed by flow cytometry. The high percentage of cell death in uninfected EB was consistent with programmed cell death during normal EB formation.[7] Therefore, EB infected by LV exhibited the same rate of cells death as control EB.

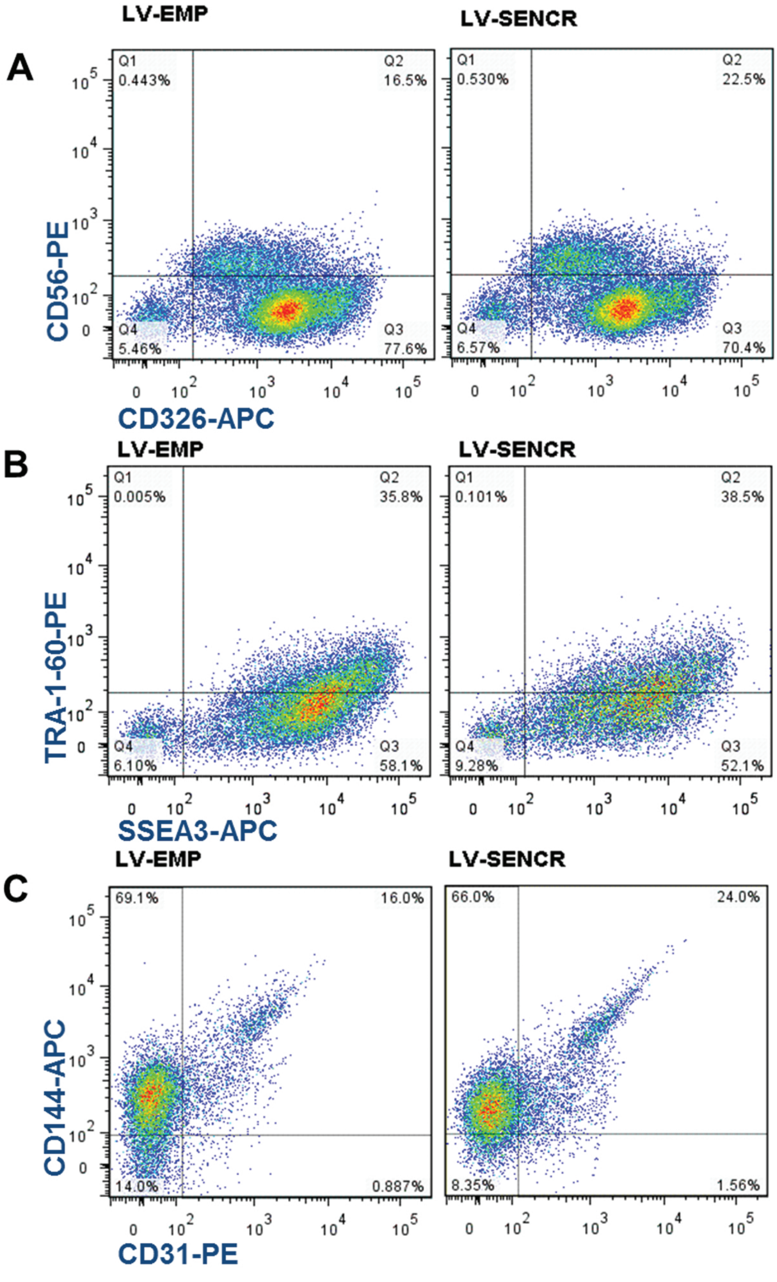

Figure S11. *SENCR* enhances differentiation of CD326^{low}CD56^{high} and CD144^{+}CD31^{+} hESC-EC. *Flow cytometry analysis of cells after SENCR overexpression: A. 3 day post-differentiation using APC-conjugated anti-CD326 and PE-conjugated anti-CD56 representing mesodermal progenitor; B. 3 day post-differentiation using APC-conjugated anti-SSEA3 and PE-conjugated anti-TRA-1-60 representing pluripotent cells; C. 7 day post-differentiation using APC-conjugated anti-CD144 and PE-conjugated anti-CD31 representing hESC-EC.*

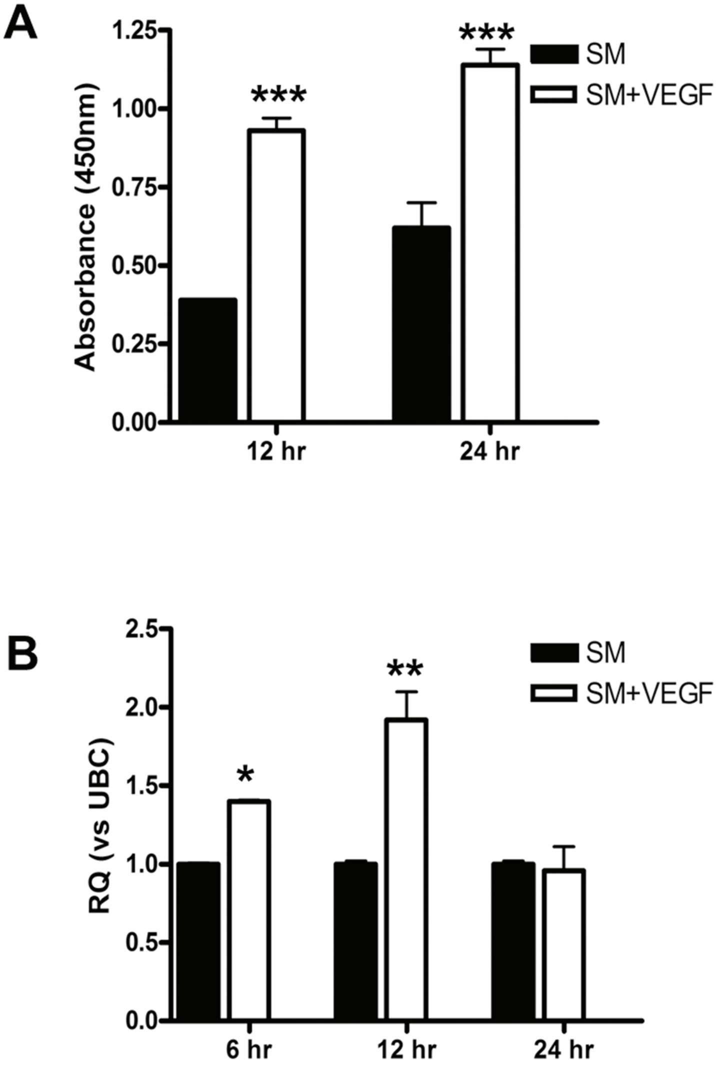

Figure S12. *SENCR* expression analysis after VEGF treatment in HUVEC. *A. Quantification of cell proliferation. HUVEC were stimulated or not with VEGF in starvation media condition (SM: control starved cells, SM+VEGF: VEGF treated cells in starved condition). After 12 and 24 h incorporated BrdU level were measured by ELISA technique and expressed as relative absorbance to untreated control. B. Quantitative expression analysis by qRT-PCR analysis showing regulation of SENCR expression in HUVEC treated with VEGF during 6, 12 and 24 h (SM + VEGF) compared to control HUVEC (SM). n=3, Student's t test, *P<0.05, **P<0.01, ***P<0.001.*

**Host**	**Isotype**	**Reactivity**	**Conjugate**	**Surface protein**
Rat	IgM	Human	Alexa Fluor 647	SSEA-3
Mouse	IgG	Human	PE	TRA1-60
Mouse	IgM	Human	APC	CD326
Mouse	IgG_1	Human	PE	CD56
Mouse	IgG_1	Human	APC	CD144
Mouse	IgG_1	Human	PE	CD31
Mouse	IgG_1	Human	FITC	CD31
Mouse	IgG_1	Human	PE-Cy™7	CD43
Mouse	IgG_1	Human	PerCP-Cy™5.5	CD117
Mouse	IgG_{2b}	Human	Brilliant Violet™ 421	CD235a
Mouse	IgG_1	Human	APC	CD73

Table S1. Information for specific antibodies used in FACS analysis and sorting.

**Host**	**Isotype**	**Reactivity**	**Conjugate**
Rat	IgM	Human	Alexa Fluor 647
Mouse	IgG	Human	PE
Mouse	IgM	Human	APC
Mouse	IgG_1	Human	PE-Cy™7
Mouse	IgG_1	Human	PerCP-Cy™5.5
Mouse	IgG_{2b}	Human	Brilliant Violet™ 421

Table S2. Information for isotype control antibodies used in FACS analysis and sorting

GENE ID	REFERENCE
UBC	HS00824723_m1
POUF51	HS03005111_g1
NANOG	HS02387400_g1
SOX2	HS01053049_s1
MESP1	HS00251489_m1
MIXL1	HS00430824
BRACHYURY	HS00610080
CD43	Hs01872322_s1
SOX17	HS00751752_s1
DKK1	HS00183740_m1
FOXA2	HS00232764_m1
SOX1	HS01057642_s1
NOTCH	HS01062014_m1
NESTIN	HS04187831_g1
GATA1	Hs00231112_m1
RUNX1	Hs00231079_m1
SCL	Hs01097987_m1
MEF2C	HS00231149_m1
ISL1	HS00158126_m1
NKX2.5	HS00231763_m1
FLT1	HS00956711_m1
CD144	HS000901463_m1
CD31	HS0016977_m1
KDR	HS0091700_m1
FLI1	HS01052961_m1
miR-27b	000409
miR-126	002228
RNU48	1006

Table S3. Information for probes used in TaqMan-based quantitative real-time PCR assays.

GENE ID	SEQUENCE
UBC	R 5'-ATC GCT GTG ATC GTC ACT TG-3'
	F 5'-TTG CCT TGA CAT TCT CGA TG-3'
GAPDH	R 5'-GCC TGC TTC ACC ACC TTC TTG-3'
	F 5'-ACA GTC CAT GCC ATC ACT GCC-3'
SENCR V1	R 5'-CCG TCT CTC CGC ATT CTC C-3'
	F 5'-TTA CCT TGT CCA CGC TCT CC-3'
SENCR V2	R 5'-GGT GGT GGA GTT GGA GTC-3'
	F 5'-GTA GAG TTT AAG CAG TGT GGA G-3'
MALAT1	R 5'-CTG GCT GCC TCA ATG CCT AC-3'
	F 5'-GTG ATG CGA GTT GTT CTC CG G-3'

Table S4. Primer-pairs used for SYBR-Green-based quantitative real-time PCR assays.

	Control<br><br>(n = 10)	Critical limb ischaemia<br><br>(n = 9)	*p* value
**Age**, years (median and IQR)	67.5 (65 to 78)	66 (60 to 79)	0.57
**Male**, n (%)	8 (80%)	7 (78%)	0.9
**Hypertension**, n (%)	9 (90%)	6 (67%)	0.21
**Diabetes**, n (%)	2 (20%)	9 (100%)	< 0.01
**Insulin therapy**, n (%)	1 (10%)	3 (33%)	0.21
**Coronary Arterial Disease**, n (%)	10 (100%)	6 (67%)	0.05
**Creatinine**, mg/dl (mean ± SEM)	1.13 ± 0.08	1.13 ± 0.20	0.98
**Chronic Renal Failure**, n (%)	3 (30%)	1 (11%)	0.31
**Statins**, n (%)	8 (80%)	3 (33%)	0.04
**Aspirin**, n (%)	9 (90%)	3 (33%)	0.01
**Clopidogrel**, n (%)	4 (40%)	4 (44%)	0.84

Table S5. Clinical characteristics of patients with or without critical limb ischaemia, from whom leftover limb muscle samples from cardiovascular surgery were analysed. Chronic renal failure defined as a creatinaemia >1.30 mg/dL. IQR = interquartile range, SEM = standard error of the mean. *P* values calculated using Mann-Witney test (for age), unpaired *t*-test (for creatinine) or the z-test for proportions (all other variables).

	Patients	Healthy Volunteers	P value
Age	51 ± 6	49 ± 5	0.37
Sex, male	8, 8 (100%)	8, 8 (100%)	0.99
**Past Medical History**			
Myocardial Infarction	5 (63%)	0 (0%)	0.01
Angina	8 (100%)	0 (0%)	<0.001
PCI	6 (75%)	0 (0%)	0.003
CABG	2 (25%)	0 (0%)	0.17
**Risk Factors**			
Current smokers	0 (0%)	0 (0%)	0.99
Hypertension	4 (50%)	1 (13%)	0.08
Hyperlipidaemia	7 (88%)	2 (25%)	0.01
**Hemodynamic Variables**			
Systolic blood pressure, mmHg	144 ± 18	138 ± 22	0.57
Diastolic blood pressure, mmHg	87 ± 12	82 ± 13	0.42
Heart rate, bpm	56 ± 9	65 ± 11	0.11
Total Cholesterol, mmol/l	5.5 ± 1.8	5.1 ± 0.8	0.59
LDL Cholesterol, mmol/l	3.6 ± 1.5	2.9 ± 1.0	0.32
Glucose (random), mmol/l	4.8 ± 0.5	4.9 ± 0.3	0.63
**Drug Treatment**			
Aspirin	8 (100%)	0 (0%)	<0.001
Clopidogrel	2 (25%)	0 (0%)	0.17
Beta blocker	5 (63%)	0 (0%)	0.01

Table S6. Clinical characteristics of patients with or without premature coronary artery diseases. Study subject demographics. Abbreviations; PCI= percutaneous coronary intervention, CABG= coronary artery bypass grafting, ACE = angiotensin converting enzyme, RB= receptor blocker.

Supplemental References

1. Ungrin MD, Joshi C, Nica A, Bauwens C, Zandstra PW. Reproducible, ultra high-throughput formation of multicellular organization from single cell suspension-derived human embryonic stem cell aggregates. *PLoS One*. 2008;3(2):e1565. doi:10.1371/journal.pone.0001565.

2. MOUNTFORD J, OLIVER E. Erythroid Production. 2014.

3. Demaison C, Parsley K, Brouns G, et al. High-level transduction and gene expression in hematopoietic repopulating cells using a human immunodeficiency [correction of imunodeficiency] virus type 1-based lentiviral vector containing an internal spleen focus forming virus promoter. *Hum Gene Ther*. 2002;13(7):803-813. doi:10.1089/10430340252898984.

4. Butler SL, Hansen MS, Bushman FD. A quantitative assay for HIV DNA integration in vivo. *Nat Med*. 2001;7(5):631-634. doi:10.1038/87979.

5. Carpentier G, Martinelli M, Courty J, Cascone I. Angiogenesis Analyzer for ImageJ. In: *4th ImageJ User & Developer Conference*. Vol ; 2012:198-201.

6. Sun X, Icli B, Wara AK, et al. MicroRNA-181b regulates NF-κB-mediated vascular inflammation. *J Clin Invest*. 2012;122(6):1973-1990. doi:10.1172/JCI61495.

7. Hernández-García D, Castro-Obregón S, Gómez-López S, Valencia C, Covarrubias L. Cell death activation during cavitation of embryoid bodies is mediated by hydrogen peroxide. *Exp Cell Res*. 2008;314(10):2090-2099. doi:10.1016/j.yexcr.2008.03.005.
